# Autophagy-dependent secretion of ENO1 mediates chemoresistance of glioblastoma and tumor microenvironment remodeling

**DOI:** 10.1038/s41419-025-08313-5

**Published:** 2025-12-06

**Authors:** Qijun Xie, Lei Chen, Yifeng Huang, Ziyuan Yu, Rongzhang Zhu, Junjie Li, Jiakun Zhao, Yiqi Song, Hong Li, Yuntao Lu

**Affiliations:** 1 Department of Neurosurgery, Nanfang Hospital, Southern Medical University, Guangzhou, China; 2 Nanfang Glioma Center, Guangzhou, China; 3 Institute of Brain Diseases, Nanfang hospital, Southern Medical University, Guangzhou, China

**Keywords:** Cancer microenvironment, CNS cancer, Prognostic markers, Cancer therapeutic resistance, Cancer metabolism

## Abstract

Acquired therapeutic resistance in glioblastoma multiforme (GBM) constitutes a major determinant of its refractory and tumor recurrence. Both tumor-intrinsic epigenetic regulation and tumor microenvironment (TME) remodeling are now understood to play pivotal roles in this resistance; however, the synergistic mechanisms and key molecular mediators underlying this interplay remain poorly defined. In this study, we demonstrated that temozolomide (TMZ) could activate the autophagy-dependent secretory pathway to promote extracellular secretion of Alpha-enolase (ENO1). Extracellular soluble ENO1 robustly enhanced GBM cell proliferation, migration, and invasion in vitro. Clinically, serum ENO1 levels were markedly elevated in GBM patients and strongly correlated with TMZ therapeutic response, suggesting its potential as a diagnostic biomarker for predicting TMZ efficacy. Mechanistically, secreted ENO1 could bind to the Toll-like receptor 4 (TLR4) receptor on GBM cells, enhancing the PI3K/Akt pathway to promote cell invasion and proliferation. Meanwhile, ENO1/TLR4 axis activated the downstream ERK/SPHK1 signaling cascade, inducing phosphorylation and membrane translocation of SPHK1 at Ser225, thereby promoting the biosynthesis of sphingosine-1-phosphate (S1P), a critical sphingolipid metabolite. Notably, extracellular ENO1 and its downstream metabolite S1P synergistically polarized tumor-associated macrophages (TAMs) toward an M2-like phenotype, fostering an immunosuppressive tumor microenvironment (TME) and conferring chemoresistance. Importantly, in vivo studies confirmed that combined therapy with the SPHK1 inhibitor PF-543, the TLR4 antagonist TAK-242, and TMZ synergistically suppressed tumor growth and significantly enhanced the efficacy of TMZ. Collectively, these findings reveal that ENO1 mediates intercellular crosstalk between GBM cells and M2-TAMs via autophagy-dependent secretion, thereby driving TMZ chemoresistance and functioning as an oncogene in GBM. Targeting the ENO1/TLR4 signaling axis reshapes the immune microenvironment and enhances the efficacy of TMZ, offering a promising therapeutic strategy and potential combinatorial targets for precision therapy in GBM.

## Introduction

Glioblastoma multiforme (GBM) is the most aggressive malignant tumor of the central nervous system in adults, characterized by high heterogeneity, an immunosuppressive microenvironment, and significant therapeutic resistance, leading to extremely poor patient prognosis [1, 2]. Despite multimodal interventions, including neuronavigation-assisted microsurgical resection, postoperative radiotherapy, and concurrent temozolomide (TMZ) chemotherapy, the median survival of GBM patients remains ≤15 months, with a 5-year survival rate ≤10% [3–5]. TMZ, an oral alkylating agent routinely used for GBM treatment, has become the first-line chemotherapeutic agent given its ability to penetrate the blood-brain barrier [6]. Although TMZ initially exhibits therapeutic potential, over 50% of GBM patients develop profound TMZ resistance, associated with O6-methylguanine-DNA methyltransferase (MGMT) methylation, isocitrate dehydrogenase (IDH) mutations, 1p/19q co-deletion status, and aberrant signaling cascades, ultimately leading to disease progression and mortality [7–9]. Thus, enhancing tumor cell chemosensitivity represents a critical strategy to overcome therapeutic resistance.

GBM progression and therapeutic resistance are driven by intricate crosstalk between tumor cells and the tumor microenvironment (TME). These hijacked intercellular interactions fuel cancer progression and dictate cellular fate and function [10]. Tumor-associated macrophages (TAMs) within the TME comprise heterogeneous subsets, including pro-inflammatory, anti-tumor M1-like macrophages and anti-inflammatory, pro-tumor M2-like macrophages [11]. M2-like TAMs dominate the TME, orchestrating angiogenesis, immunosuppression, metastasis, and chemoresistance, ultimately correlating with poor prognosis [12, 13]. High TAM infiltration has frequently been associated with adverse clinical outcomes across malignancies and linked to diminished responses to standard therapies, including radiotherapy, chemotherapy, and targeted agents [14]. Recent studies have highlighted the dynamic interplay between immune cells and tumor cells within the TME as a central driver of GBM progression and recurrence. Tumor cells and TAMs engage in a “malignant symbiosis” through bidirectional signaling: tumor cells secrete chemokines, metabolites, and extracellular vesicles (e.g., exosomes) to reprogram TAM function, while TAMs reciprocally promote tumor progression through growth factors, cytokines, and proteases [15]. Notably, tumor-derived secretory proteins such as cathepsin K (CTSK) can activate tumor cell migration via autocrine signaling and induce macrophage M2 polarization by binding to TLR4 receptors and stimulating mTOR-dependent pathways [16]. However, the molecular mechanisms underlying tumor cell-mediated recruitment and polarization of TAMs remain a critical unresolved challenge in cancer immunotherapy. Emerging evidence underscores the metabolic-immune crosstalk between GBM cells and TAMs as a research frontier. Elucidating the molecular basis of this crosstalk is pivotal for comprehending the heterogeneity of the immunosuppressive TME and devising strategies to overcome therapeutic limitations. This study aims to elucidate how GBM cells utilize secretory pathways to reprogram TAMs, providing novel insights into the development of combinatorial immunotherapies for GBM.

Autophagy has emerged as a pivotal player in tumorigenesis and therapy. Current evidence suggests that autophagy is upregulated during cancer therapy, enabling tumor cells to evade treatment through chemoprotective mechanisms [17]. Evidences have suggested that TMZ induces autophagy upregulation in GBM, driving chemoresistance, while autophagy inhibition enhances TMZ sensitivity [18–21]. Thus, modulating autophagy represents a promising strategy to circumvent TMZ resistance and improve therapeutic outcomes in GBM patients. Recent studies have revealed an intersection between autophagy and secretory processes; however, the role of autophagy-mediated secretion in remodeling the TME remains poorly understood. Secretory autophagy (SA) was initially identified through investigations into the extracellular release of proteins lacking canonical signal peptide sequences—which prevents their entry into the endoplasmic reticulum (ER)—a process termed unconventional protein secretion (UPS) [22, 23]. As a pathway independent of the conventional ER–Golgi trafficking system, secretory autophagy mediates the unconventional secretion of cytoplasmic cargo, such as cytosolic proteins devoid of signal peptides [24]. Unlike degradative autophagy, secretory autophagy, as a specialized cellular mechanism, selectively packages intracellular cargo (e.g., proteins, cytokines, pathogens) into autophagosomes, which subsequently fuse with the plasma membrane to release their contents into the extracellular space. This process facilitates intercellular communication, immune modulation, and pathological remodeling within the TME [25].

Alpha-enolase (ENO1), a key glycolytic enzyme, functions as a multifunctional oncoprotein with critical roles in tumorigenesis [26]. Recent studies have revealed that ENO1 not only drives metabolic reprogramming in cancer cells but also acts as a moonlighting protein, actively secreted into the TME via exosomes or in soluble form, thereby regulating intercellular communication and immune evasion [27]. For instance, exosomal ENO1 transferred between hepatocellular carcinoma (HCC) cells mimics the pro-metastatic effects of endogenous ENO1 overexpression. Exosome-derived ENO1 upregulates integrin α6β4 expression and activates the FAK/Src-p38MAPK signaling axis, thereby enhancing the growth and metastasis of ENO1-low HCC cells [28]. In oral squamous cell carcinoma (OSCC), ENO1 synergizes with macrophage-derived IL-6 through lactate secretion and extracellular protein interactions to promote tumor cell migration, invasion, and epithelial-mesenchymal transition (EMT), establishing a feedforward loop that accelerates OSCC progression [29]. These findings collectively suggest ENO1 as a potential mediator of GBM-TAM crosstalk.

Although ENO1 overexpression in GBM is correlated with a poor prognosis and drives malignant progression, its extracellular roles in glioblastoma remain notably unexplored. Specifically, whether GBM cells employ autophagy-dependent secretion of ENO1 to orchestrate symbiotic interactions with TAMs within the TME remains elusive. Herein, the molecular mechanism by which TMZ-induced autophagy-dependent secretion of ENO1 exacerbates chemoresistance was explored. The mechanism was found to involve autocrine signaling and M2-like TAM polarization. Our study aims to unravel how ENO1-mediated intercellular crosstalk reshapes the immunosuppressive TME and sustains TMZ resistance in GBM.

## Results

### Temozolomide promotes ENO1 secretion of GBM cells through unconventional secretory pathways

To determine whether TMZ enhances autophagic activity in glioblastoma, U87MG cells were treated with 600 μM TMZ for 48 h. Immunofluorescence analysis revealed a significant increase in LC3B-positive puncta with characteristic dot-like aggregation in TMZ-treated cells (Supplementary Fig. 1A). Western blot demonstrated enhanced conversion of LC3B-I to LC3B-II (Supplementary Fig. 1B), while the typical double-membrane autophagosome-like vesicles increased in TMZ-treated cells compared to the control cells under transmission electron microscopy (TEM) (Supplementary Fig. 1C). These results collectively indicate that TMZ treatment significantly activates autophagic flux in GBM cells.

**Fig. 1:**
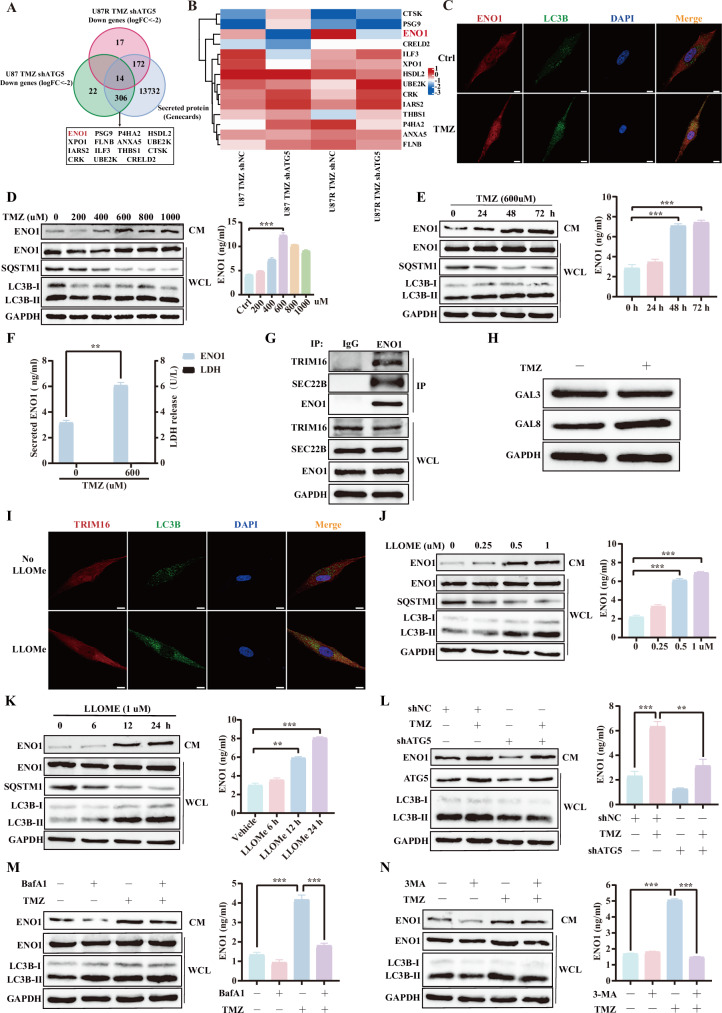
Temozolomide induces autophagy-dependent ENO1 secretion in glioblastoma. **A** Secretome profiling via 4D-FastDIA proteomics and GeneCards database screening identified secreted proteins. **B** Heatmap analysis of the identified secreted proteins. **C** Immunofluorescence (IF) staining demonstrating ENO1 colocalization with autophagy marker LC3B. Scale bars, 20 μm. **D** Western blot and ELISA analysis of secreted ENO1 levels in conditioned media from GBM cell lines exposed to TMZ (0–1000 μM, 48 h). **E** Time-course analysis (fixed 600 μM TMZ) monitoring ENO1 secretion by Western blot and ELISA. **F** Extracellular ENO1 quantification by ELISA and membrane integrity assessment via LDH release assay in 600 μM TMZ-treated models. **G** Co-immunoprecipitation (Co-IP) of GBM cell lysates using anti-ENO1 antibody, followed by immunoblotting with anti-TRIM16 and anti-SEC22B antibodies. **H** Western blot analysis of GAL3 and GAL8 expression in GBM cells treated with 600 μM TMZ. **I** IF double-labeling showing LLOMe (0.5 μM)-induced subcellular colocalization of LC3B and TRIM16. Scale bars, 20 μm. **J** Dose-response analysis: GBM cells treated with LLOMe (0, 0.25, 0.5, 1 μM; 24 h). Intracellular ENO1, LC3B-I-to-II conversion (Western blot), and secreted ENO1 (ELISA) were concurrently measured. **K** Time-course analysis (fixed 1 μM LLOMe; 0, 6, 12, 24 h): Intracellular ENO1/LC3B (Western blot) and secreted ENO1 (ELISA). **L** ATG5-knockdown validation: shNC/shATG5-transfected GBM cells ± TMZ (600 μM, 24 h). Immunoblotting for ATG5, secreted ENO1, and LC3B lipidation; secreted ENO1 quantified by ELISA. **M** Bafilomycin A1 (BafA1, 200 nM, 6 h pre-treatment) ± TMZ (600 μM, 24 h). Immunoblotting of whole-cell lysates (WCL) and conditioned media (CM) for LC3B/ENO1; secreted ENO1 by ELISA. **N** 3-Methyladenine (3-MA, 5 mM, 6 h pre-treatment) ± TMZ (600 μM, 24 h). Immunoblotting of WCL and CM for LC3B/ENO1; secreted ENO1 by ELISA. Data are expressed as mean ± SEM. ns not significant, *P < 0.05, **P < 0.01, ***P < 0.001.

To evaluate the role of ATG5-mediated autophagy-dependent secretion in regulating malignant phenotypes, ATG5-knockdown U87 cells (shATG5) were established using lentiviral transduction (Supplementary Fig. 1D). Conditioned media from TMZ-treated shNC cells significantly enhanced glioblastoma cell proliferation and migration compared to media derived from TMZ-treated shATG5 cells (Supplementary Fig. 1E, F), suggesting that TMZ induces ATG5-dependent secretion of specific factors that remodel tumor behavior. To investigate TMZ-induced secretory autophagy and comprehensively characterize the released cargo proteins, 4D-FastDIA quantitative proteomics was conducted on conditioned media from TMZ-treated shATG5 U87 and TMZ-resistant U87-TMZR cells, yielding 14 consistently downregulated secreted proteins (logFC < −2) compared to TMZ-treated shNC controls (Fig. 1A). Among these, ENO1 was identified as the most significantly downregulated protein in the secretomes of both TMZ-shATG5 and TMZR-shATG5 cells (Fig. 1B), and its function had not been previously explored in the extracellular environment of gliomas.

To determine whether ENO1 secretion depends on the classical ER-Golgi secretory pathway, we treated cells with Brefeldin A, an inhibitor of ER-to-Golgi trafficking. Notably, while Brefeldin A treatment nearly abrogated secretion of the positive control FN1, it yielded no inhibitory effect on endogenous ENO1 secretion (Supplementary Fig. 2A). This observation was further supported by SignalP analysis, which confirmed the absence of a canonical secretion signal peptide in ENO1 (Supplementary Fig. 2B). Collectively, these results definitively exclude the involvement of classical secretory pathways in ENO1 secretion, indicating that ENO1 secretion occurs via non-classical mechanisms.

Confocal microscopy revealed TMZ-induced colocalization of ENO1 with LC3B-positive puncta (Fig. 1C), suggesting ENO1 as a potential autophagic cargo. Dose- and time-course experiments showed TMZ (peaking at 600 μM, 48 h) simultaneously enhanced LC3B-II conversion with a corresponding dose- and time-dependent extracellular ENO1 secretion (Fig. 1D, E). To exclude the possibility of passive ENO1 release through membrane disruption, lactate dehydrogenase (LDH) release, a cytoplasmic integrity marker, was quantified in TMZ-treated cells (600 μM, 48 h). Importantly, the absence of LDH release confirmed this secretory process was not attributable to membrane permeabilization (Fig. 1F). These findings collectively demonstrate that TMZ promotes ENO1 secretion through an unconventional, autophagy-dependent pathway.

The molecular machinery modulating secretory autophagy involves TRIM16, a selective cargo receptor, and the vesicular transporter SEC22B, which collectively form a core regulatory module. Canonical secretory autophagy cargoes such as IL-1β are recognized by TRIM16, which subsequently recruits the R-SNARE protein SEC22B to facilitate IL-1β release into the extracellular space [30]. To investigate the unconventional secretion of ENO1, we examined its interactions with TRIM16 and SEC22B, key components of the unconventional protein secretion (UPS) pathway, and characterized their interactions with ENO1. Co-immunoprecipitation assays demonstrated ENO1 interaction with TRIM16 (cargo receptor) and SEC22B (vesicular transporter) (Fig. 1G). Compromised lysosomal integrity has been established as a hallmark of secretory autophagy activation [31]. To evaluate whether TMZ-triggered secretory autophagy involves lysosomal destabilization, we monitored the expression of galectin-3 (GAL3) and galectin-8 (GAL8), established markers of endomembrane damage that signal through TRIM16 complex recruitment [32, 33]. Notably, TMZ specifically upregulated GAL8 (Fig. 1H), a lysosomal damage marker. Moreover, LLOMe-induced lysosomal stress (a canonical secretory autophagy inducer [34]) promoted both TRIM16-LC3B colocalization (Fig. 1I) and ENO1 secretion concomitant with LC3B-II accumulation (Fig. 1J, K). These findings position TRIM16 as a molecular link between lysosomal stress and autophagy-dependent ENO1 secretion. Further experiments revealed that ATG5 knockdown attenuated both LC3B lipidation and TMZ-induced ENO1 secretion (Fig. 1L). While TMZ alone increased LC3B-II (indicating autophagic flux), combined treatment with autophagy inhibitors (BafA1 or 3-MA) suppressed ENO1 secretion (Fig. 1M, N), suggesting that autophagic flux activation, rather than impaired autophagosomal degradation, mediates ENO1 secretion. These findings establish that TMZ activates an autophagy-dependent secretory pathway to drive ENO1 release in GBM.

### ENO1 overexpression correlates with glioma progression and TMZ resistance

**Fig. 2:**
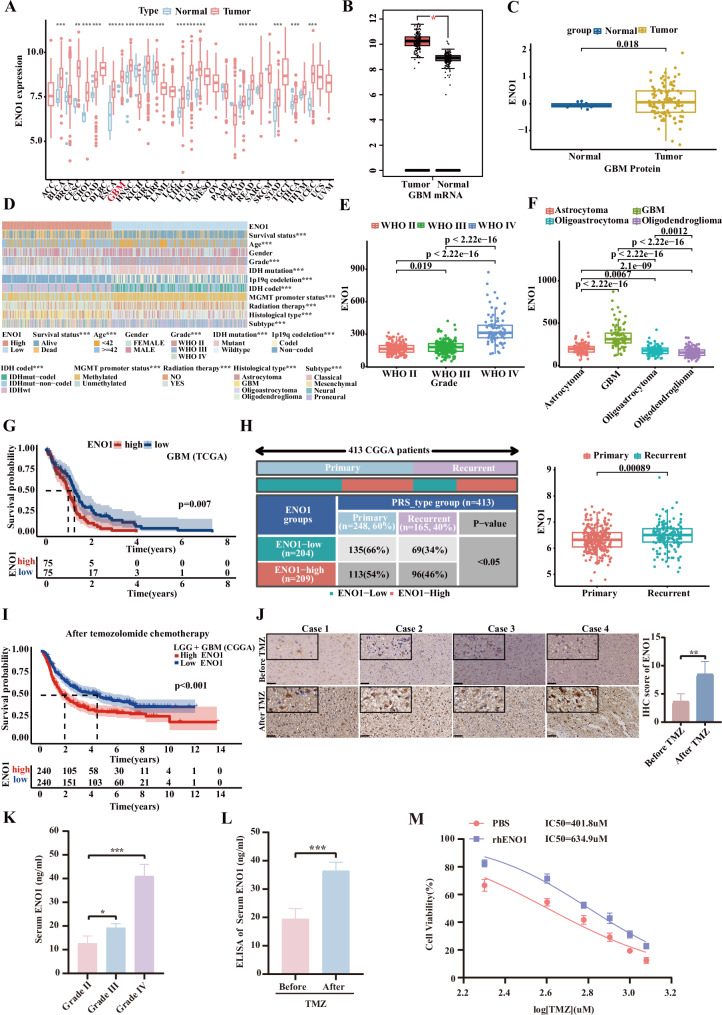
ENO1 overexpression correlates with glioma progression and TMZ resistance. **A** Pan-cancer analysis of ENO1 expression. ENO1 expression in normal brain tissues and GBM tumor tissues at mRNA (**B**) and protein levels (**C**). **D** Heatmap analysis correlating ENO1 expression with clinicopathological features in glioma patients. Significant associations between ENO1 expression and WHO grading (**E**) or histopathological subtypes (**F**). **G** Kaplan-Meier overall survival (OS) analysis of TCGA-GBM patients stratified by high/low ENO1 mRNA expression. **H** Analysis of mRNA expression levels of ENO1 in patients with primary recurrent gliomas based on the CGGA database. **I** Overall survival (OS) analysis of TMZ-treated glioma patients stratified by ENO1 expression based on the CGGA-LGG + GBM database. **J** Representative IHC images and quantitative scores of ENO1 in paired primary/recurrent GBM specimens pre- and post-TMZ therapy. Scale bars, 50 μm. **K** Serum ENO1 levels quantified by ELISA across glioma WHO grades. **L** ELISA quantitative analysis of serum ENO1 concentrations in GBM patients pre- and post-TMZ chemotherapy. **M** TMZ sensitivity assays following exogenous rhENO1 (1 μg/mL) administration, measured by IC50 shifts. Data are expressed as mean ± SEM. ns not significant, *P < 0.05, **P < 0.01, ***P < 0.001.

To elucidate the clinicopathological relevance of secretory ENO1, its association with glioma features was systematically analyzed using TCGA database. Compared to normal brain tissues, GBM showed significantly elevated ENO1 expression at both mRNA and protein levels (Fig. 2A–C). ENO1 expression positively correlated with WHO glioma grade (P < 0.05) and varied significantly among histological subtypes, with particularly high expression in glioblastoma (Fig. 2D–F). Molecular subtyping revealed that ENO1 was preferentially expressed in IDH wild-type, 1p19q non-codeleted, MGMT promoter unmethylated, and mesenchymal subtype tumors compared to their counterparts (*P* < 0.05, Supplementary Fig. 3A–F). Survival analysis further demonstrated that GBM patients with high ENO1 expression exhibited significantly worse clinical outcomes than those with low expression (*P* < 0.05, Fig. 2G). Analysis of 413 primary and recurrent glioma cases from the CGGA database showed significantly higher ENO1 mRNA levels in recurrent cases (*P* < 0.05, Fig. 2H). Among TMZ-treated patients in CGGA cohort, elevated ENO1 expression correlated with poorer prognosis (*P* < 0.05, Fig. 2I). In our longitudinal study of 12 GBM patients receiving the standard Stupp regimen (postoperative radiotherapy with concurrent TMZ chemotherapy), we analyzed matched pre-/post-treatment peripheral blood samples (EDTA-anticoagulated, n = 12) and tissue specimens (n = 12). Immunohistochemistry revealed increased ENO1 protein expression and IHC scores in recurrent tumors after TMZ chemotherapy (Fig. 2J). ELISA confirmed elevated serum ENO1 levels in GBM patients, with further increase observed post-TMZ treatment (Fig. 2K, L). Subsequent exogenous administration of recombinant human ENO1 (rhENO1) increased the IC50 of TMZ (Fig. 2M), demonstrating its functional role in reducing TMZ sensitivity and promoting a resistance phenotype. These findings establish extracellular ENO1 accumulation as a TMZ-induced stress response that correlates with therapeutic resistance, positioning it as a promising biomarker for precision GBM therapy.

### Extracellular soluble ENO1 promotes proliferation, migration and invasion of glioblastoma cells

**Fig. 3:**
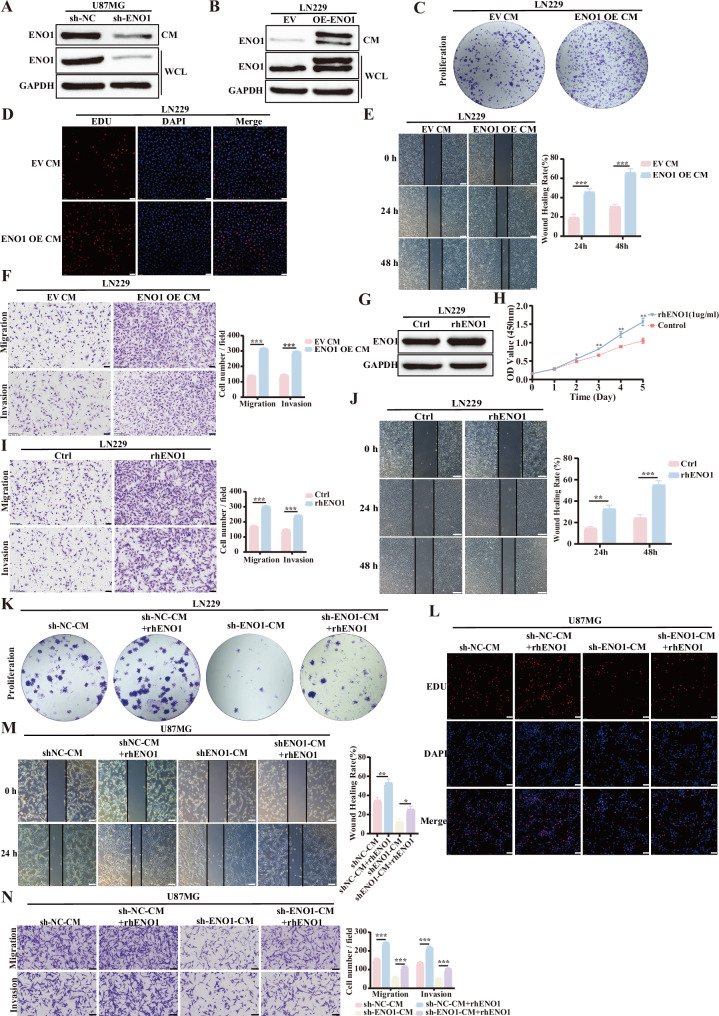
Soluble ENO1 promotes proliferation, migration, and invasion of GBM cells in vitro*.* **A** Western blot analysis of ENO1 knockdown efficiency in both intracellular and extracellular fractions of U87MG cells. **B** Western blot validation of ENO1 overexpression in LN229 cells and conditioned media (CM). Colony formation (**C**) and EdU proliferation (**D**) assays of parental LN229 cells treated with CM from ENO1-overexpressing LN229 cells. Scale bars, 100 μm. **E**, **F** Metastatic potential assessment using CM from ENO1-overexpressing cells: **E** Wound healing assay (scale bar = 500 μm). **F** Transwell migration and invasion assays (scale bar = 100 μm). **G** Western blot detection of intracellular ENO1 after 24 h treatment with recombinant human ENO1 (rhENO1, 1 μg/mL). **H** CCK-8 assay evaluating proliferation in rhENO1-treated cells (1 μg/mL). **I** Transwell migration/invasion of rhENO1-treated GBM cells (scale bars, 100 μm). **J** Scratch wound healing assay post-rhENO1 treatment (scale bars, 500 μm). Rescue experiments: Proliferation (**K**, **L**) and migration/invasion (**M**, **N**) capacities of GBM cells treated with shENO1 CM supplemented with rhENO1. Data are expressed as mean ± SEM. ns not significant, *P < 0.05, **P < 0.01, ***P < 0.001.

Analysis of the Cancer Cell Line Encyclopedia (CCLE) database revealed significant upregulation of ENO1 expression in most glioma cell lines (Supplementary Fig. 3G). To validate these findings, we performed Western blot analysis on six glioma cell lines, which confirmed varying degrees of ENO1 upregulation (Supplementary Fig. 3H). Based on their endogenous expression level, U87MG (ENO1 high) and LN229 (ENO1 low) were selected to establish ENO1-knockdown and overexpressing cell models, respectively (Supplementary Fig. 3I, J). To investigate potential intercellular communication mediated by secretory ENO1 within the TME, we analyzed serum-free conditioned medium (CM) and whole-cell lysates (WCL) from genetically modified GBM cells. Western blot results demonstrated reduced intracellular ENO1 protein levels and diminished extracellular secretion in *shENO1* groups compared to controls (Fig. 3A), whereas OE-ENO1 groups exhibited marked upregulation of ENO1 in both compartments **(**Fig. 3B). To further evaluate the functional impact of extracellular soluble ENO1 on GBM cell proliferation, migration, and invasion, CM intervention models were established using ENO1-knockdown U87MG and ENO1-overexpressing LN229 cells. ENO1-ovexpressing CM significantly enhanced these malignant phenotypes (Fig. 3C–F). Exogenous administration of rhENO1 recapitulated the oncogenic effects observed with ENO1 overexpression (Fig. 3G–J). Notably, rhENO1 treatment partially rescued the shENO1-mediated suppression of tumorigenic potential, restoring GBM cell proliferation, migration, and invasion capabilities (Fig. 3K–N).

### TLR4 receptor is essential for ENO1-mediated malignant progression in glioblastoma

**Fig. 4:**
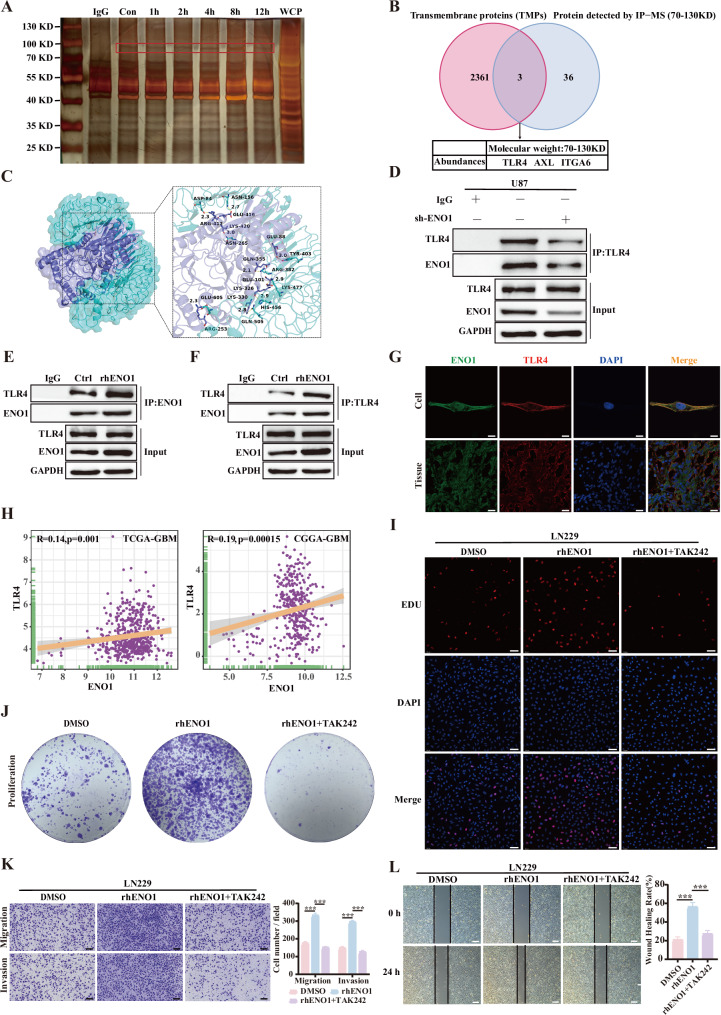
TLR4 receptor is essential for ENO1-mediated malignant progression in glioblastoma. **A** Immunoprecipitation-mass spectrometry (IP-MS) analysis of ENO1-interacting proteins in U87MG cells treated with rhENO1 (0-12 h) using ENO1-conjugated beads, with whole cell protein (WCP) and IgG as controls (silver staining shown). **B** Identification of ENO1-binding transmembrane proteins (TMPs) from 70-130 kDa differential bands by MS, screened against the Membranome database (containing 2364 TMPs). **C** Molecular docking model of ENO1-TLR4 interaction (ENO1 shown in deep blue, TLR4 in cyan; binding sites displayed as stick structures in corresponding colors). **D**–**F** Co-immunoprecipitation (Co-IP) validation of ENO1-TLR4 interaction in GBM cells: **D** Co-IP validated that ENO1 binds to TLR4 in shENO1 GBM cells. **E** Co-IP with anti-ENO1 antibody followed by immunoblotting of rhENO1-treated GBM cells with anti-ENO1 and anti-TLR4 antibodies. **F** Co-IP with anti-TLR4 antibody followed by immunoblotting of rhENO1-treated GBM cells with anti-ENO1 and anti-TLR4 antibodies. **G** Immunofluorescence analysis of ENO1 (green) and TLR4 (red) colocalization in rhENO1-stimulated GBM cells and TMZ-treated human GBM specimens. Scale bar = 10 μm. **H** Pearson correlation analysis of ENO1 and TLR4 mRNA expression in TCGA-GBM and CGGA-GBM datasets. Representative images and quantitative analysis of (**I**) proliferation, (**J**) colony formation, and migration/invasion (**K**, **L**) in GBM cells treated with or without TLR4 inhibition and cultured in rhENO1. Scale bars = 5 mm (**J**), 100 μm (**I**, **K**), and 500 μm (**L**). Data are expressed as mean ± SEM. ns not significant, *P < 0.05, **P < 0.01, ***P < 0.001.

Our findings demonstrated that soluble ENO1 could drive GBM malignancy by regulating tumor cell proliferation, migration, and invasion through an autophagy-dependent secretory pathway. However, the specific cellular receptor and downstream signaling mechanisms of ENO1 remained unclear. Accordingly, U87MG cells were treated with rhENO1 (1 μg/mL) for 0, 1, 2, 4, 8, or 12 h. Subsequent immunoprecipitation (IP) using ENO1-specific antibody-conjugated beads and silver staining of whole-cell lysates revealed enhanced protein binding in the 70–130 kDa range (Fig. 4A), suggesting the presence of functional ENO1-interacting proteins. Differential bands in this region were excised for gel purification and mass spectrometry (IP-MS), identifying 39 candidate interactors. To identify ENO1-specific membrane receptors, we cross-referenced these results with the Membranome database (2,364 transmembrane proteins), yielding three candidates: TLR4, AXL, and ITGA6 (Fig. 4B). While prior studies have implicated extracellular ENO1 in CD14-dependent TLR4 signaling in monocytes [27] and a paracrine ENO1/TLR4/IL-6 axis driving metastasis in oral squamous cell carcinoma [29], its direct interaction with TLR4 in GBM remained unexplored. Structural modeling using PyMOL (ENO1: PDB ID 2PSN; TLR4: PDB ID 8WO1) predicted hydrogen bonds between ENO1 residues (K330, K326, R412, Q355, K420, E88, E416, E101, R253) and TLR4 residues (Q505, H456, D84, R382, N265, Y403, N156, K477, E605) (Fig. 4C). Co-IP assays using shENO1 cells confirmed the interaction between ENO1 and TLR4 in GBM cells, with diminished binding upon ENO1 knockdown (Fig. 4D). Co-IP further validated that rhENO1 (1 μg/mL) increased binding between ENO1 and TLR4 in GBM cells (Fig. 4E, F), and immunofluorescence (IF) assays demonstrated their co-localization in GBM cells and tissues (Fig. 4G).

TCGA-GBM and CGGA-GBM datasets revealed a positive correlation between ENO1 and TLR4 expression (Fig. 4H). Functional assays with the TLR4 inhibitor TAK-242 demonstrated that TLR4 blockade reversed rhENO1-induced tumor proliferation, migration, and invasion (Fig. 4I–L), confirming TLR4’s role in ENO1-driven malignancy. Similarly, TLR4 knockdown via lentiviral shRNA (Supplementary Fig. 4A, B) attenuated the pro-tumorigenic effects of ENO1-overexpressing conditioned medium (OE-ENO1 CM) on GBM cell phenotypes (Supplementary Fig. 4C–F). These findings collectively establish cell membranous ENO1-TLR4 receptor interaction as a critical axis in GBM progression.

### ENO1 promotes GBM malignant progression partially via activating the PI3K/AKT signaling pathway

**Fig. 5:**
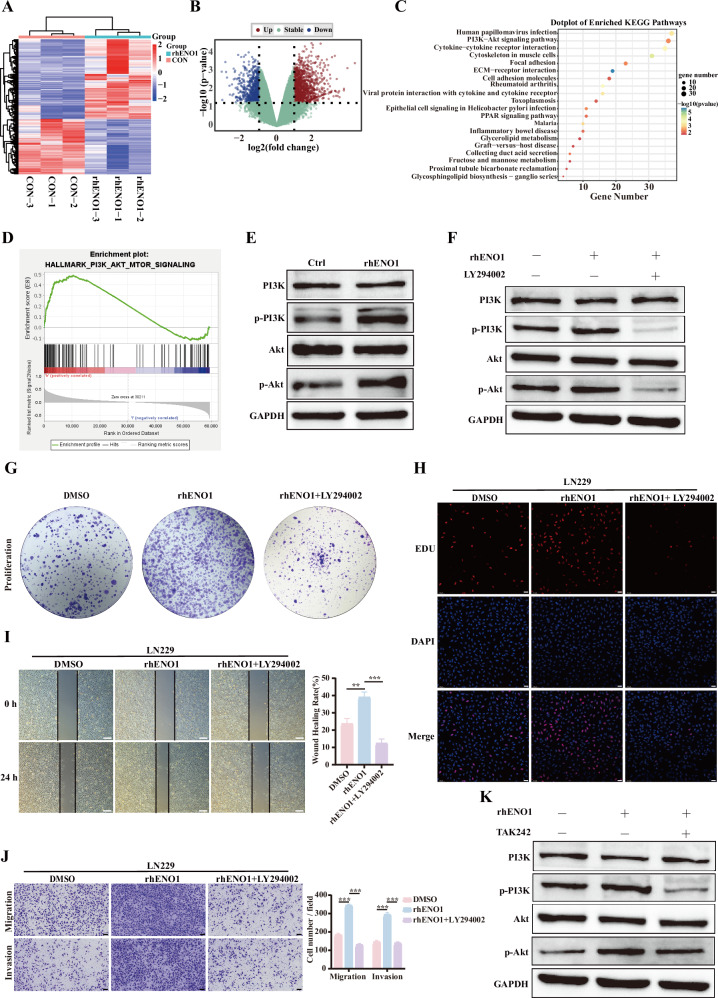
ENO1 drives GBM malignant progression through PI3K/AKT signaling activation. **A** Cluster analysis of differentially expressed genes (DEGs) between rhENO1-treated and control groups (n = 3 biological replicates). **B** Volcano plot of DEGs displaying: X-axis: log2 fold-change in gene expression; Y-axis: -log10(p-value) for statistical significance; Green dots: non-significant genes; Red/blue dots: significantly up-/down-regulated genes. **C** KEGG pathway analysis revealing significant association between ENO1 and PI3K- Akt signaling in GBM patient samples. **D** GSEA enrichment plot demonstrating PI3K- Akt pathway activation correlated with ENO1 expression from the TCGA database. **E** Western blot analysis of PI3K and Akt phosphorylation in rhENO1-treated GBM cells using anti-phospho-PI3K, anti-PI3K, anti-phospho- Akt, and anti- Akt antibodies. **F** Western blot analysis of PI3K/Akt phosphorylation in serum-starved GBM cells treated with/without rhENO1 (1 μg/mL) and PI3K inhibitor LY294002. Representative images and quantitative analysis of **G** colony formation, **H** proliferation, and migration/invasion **I**, **J** in GBM cells treated with or without PI3K inhibition LY294002 and cultured in rhENO1. Scale bars = 5 mm (**G**), 100 μm (**H, J**), and 500 μm (**I**). **K** Western blot analysis of PI3K/Akt phosphorylation in serum-starved GBM cells treated with/without rhENO1 (1 μg/mL) and TLR4 inhibitor TAK242 (10 μM). Data represent mean ± SEM; ns: not significant, *P < 0.05, **P < 0.01, ***P < 0.001.

While prior studies have established that ENO1 binds TLR4 on GBM cells to regulate malignancy, its downstream signaling cascades remained unexplored. Existing evidence indicates that constitutively elevated ENO1 activates FAK/PI3K/Akt signaling [35]. To delineate how secreted ENO1 sustains malignant phenotypes, we performed RNA-seq analysis of rhENO1-stimulated GBM cells (Fig. 5A, B). KEGG pathway enrichment revealed significant association between ENO1 and PI3K/Akt signaling (Fig. 5C, D). Western blot confirmed rhENO1 increased p-PI3K/PI3K and p-Akt/Akt ratios, indicating pathway activation (Fig. 5E). To validate the functional importance of PI3K/Akt signaling, GBM cells were treated with rhENO1 in the presence or absence of PI3K inhibitor LY294002. Western blot analysis confirmed that LY294002 effectively suppressed rhENO1-induced phosphorylation of PI3K and Akt (Fig. 5F). Functional assays demonstrated that LY294002 partially attenuated rhENO1-driven cell proliferation, migration and invasion capacities (Fig. 5G–J). Consistently, TAK-242-mediated TLR4 inhibition attenuated PI3K-Akt activation (Fig. 5K), suggesting ENO1 may activate additional signaling cascades beyond PI3K/Akt to contribute to GBM aggressiveness. Compared with the effects of TAK-242 (Fig. 4J–L), the PI3K/AKT inhibitor only partially attenuated TLR4-mediated effects, whereas TLR4 inhibition substantially blocked rhENO1-induced responses, suggesting the involvement of additional downstream mechanisms of TLR4 signaling. To investigate the impact of PI3K/AKT signaling on tumor formation, we conducted in vivo brain tumor experiments using luciferase-labeled GL261 cells and monitored tumor growth via bioluminescence imaging (BLI) (Supplementary Fig. 5A). Consistent with in vitro results, BLI showed that TMZ + LY294002 and TMZ + LY294002 + TAK-242 all inhibited brain tumor growth compared to TMZ treatment only, with the triple combination showing the strongest inhibitory effect (Supplementary Fig. 5B–D). Survival analysis revealed that all treatment groups prolonged the survival time of the mice, with the TMZ + LY294002 + TAK-242 group demonstrating the longest survival (Supplementary Fig. 5E). Subsequent IHC staining showed significantly reduced expression of ENO1, p-PI3K, and p-AKT in tumor tissues from the combination therapy groups (Supplementary Fig. 5F). Our results highlight the crucial role of the TLR4-PI3K/AKT signaling pathway in regulating GBM malignant progression and provide new therapeutic possibilities.

### Soluble ENO1 drives malignant phenotypes in glioblastoma via TLR4-mediated activation of the SPHK1-S1P signaling cascade

To investigate alternative mechanisms, we focused on the glycolytic function of ENO1. As a critical metabolic hub in glycolysis, the non-canonical functions of ENO1 have attracted significant interest. Recent studies suggest its potential involvement in other cellular metabolic processes. Sun and colleagues first established the ENO1*-*YAP1/PLCB1/HPGD regulatory axis, demonstrating its glycolytic-independent mechanism in promoting tumor growth via arachidonic acid (AA) metabolic reprogramming. This study innovatively revealed ENO1 as a metabolic cross-regulatory node [36]. Similarly, Ma et al. reported that ENO1 facilitates GBM choline phospholipid metabolism and tumor cell proliferation through its moonlighting functions, highlighting its integrated regulation of cancer metabolism via crosstalk between glycolysis and phospholipid synthesis [37]. These findings confirm ENO1’s roles beyond glycolysis, particularly in lipid metabolism pathways such as AA and choline phospholipid metabolism. To further investigate ENO1*-*associated non-glycolytic metabolic pathways in tumor progression, we analyzed proteomic profiles of 110 GBM patients from the Clinical Proteomic Tumor Analysis Consortium (CPTAC) database. Gene Set Variation Analysis (GSVA) and Gene Set Enrichment Analysis (GSEA) revealed that the ENO1-high group exhibited significant enrichment not only in glycolysis (NES = 2.03, *p* < 0.001) but also in lipid metabolism (NES = 1.62, *p* < 0.001) and phospholipid metabolism (NES = 1.35, *p* = 0.01) pathways (Supplementary Fig. 6A, B). These results suggest that ENO1 is linked to non-glycolytic metabolic pathways in GBM, especially phospholipid and lipid metabolism.

**Fig. 6:**
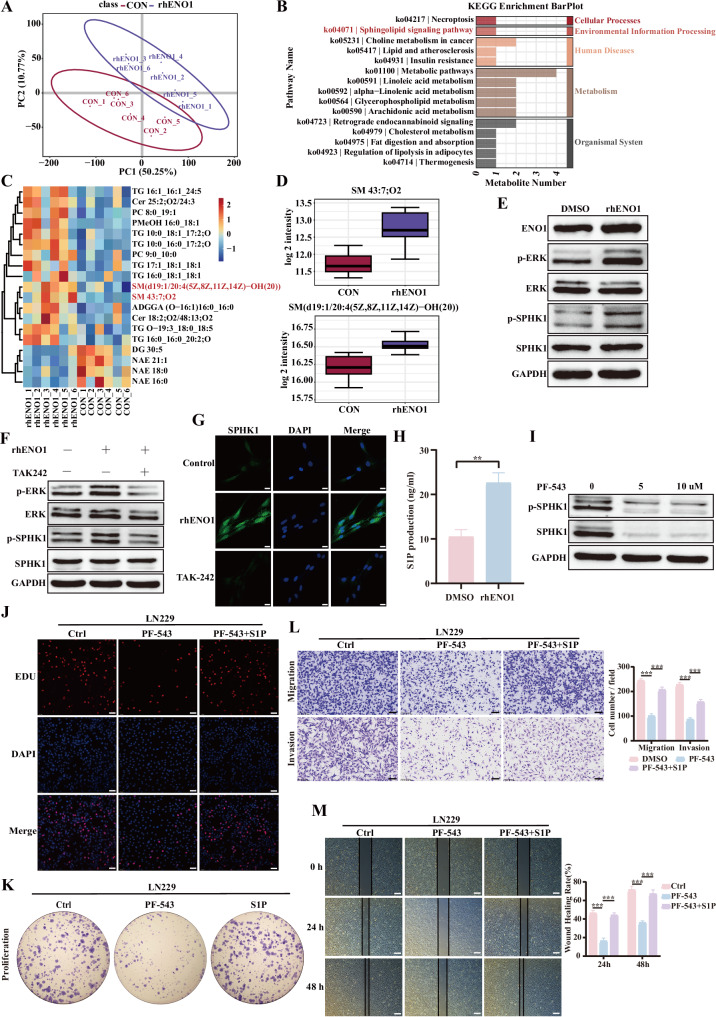
Soluble ENO1 drives malignant phenotypes in glioblastoma through TLR4-mediated activation of the SPHK1-S1P signaling cascade. **A** Principal component analysis (PCA) of metabolites and lipids detected by untargeted lipidomics in GBM cells treated with soluble recombinant ENO1 (rhENO1, 1 μg/mL) (n = 6 biological replicates). **B** KEGG pathway enrichment analysis of differential metabolites with VIP > 1. **C** Heatmap analysis of differential metabolites with VIP > 1 (n = 6 biological replicates). **D** Sphingomyelin (SM) was significantly upregulated in rhENO1-treated samples. **E** Western blot analysis of SPHK1 and ERK phosphorylation levels in serum-starved GBM cells treated with or without rhENO1 (1 μg/mL) for 6 h. **F** Western blot analysis using anti-phospho-SPHK1, anti-SPHK1, anti-phospho-ERK, and anti-ERK antibodies in serum-starved GBM cells treated with or without rhENO1 (1 μg/mL) and TLR4 inhibitor (TAK242, 10 μM) for 6 h. **G** Immunofluorescence staining of SPHK1 subcellular localization in GBM cells treated with rhENO1 (1 μg/mL) and TLR4 inhibitor (TAK242, 10 μM). Scale bar = 10 μm. **H** ELISA quantification of S1P levels in conditioned medium from rhENO1-treated GBM cells. **I** Western blot analysis of SPHK1 phosphorylation levels in GBM cells treated with increasing concentrations of SPHK1 inhibitor PF-543. J-M Representative images and quantification of **J** proliferation (scale bar = 100 μm), **K** colony formation (scale bar = 5 mm), **L** migration (scale bar = 100 μm), and **M** invasion (scale bar = 500 μm) in GBM cells treated with DMSO control, PF-543 (10 μM), or S1P (1 μM). Data are expressed as mean ± SEM. ns not significant, *P < 0.05, **P < 0.01, ***P < 0.001.

To elucidate how extracellular soluble ENO1 sustains GBM malignancy, we performed untargeted lipid metabolomics using U87MG cells treated with rhENO1 (1 μg/mL) via LC-MS/MS. Principal component analysis (PCA) demonstrated a clear separation between rhENO1-treated and control groups based on metabolite abundance (Fig. 6A). Nineteen differentially abundant metabolites (VIP > 1, *P* < 0.01) were identified, with KEGG pathway analysis highlighting alterations in sphingolipid signaling (Fig. 6B). Hierarchical clustering identified sphingomyelin (SM) as the most significantly upregulated lipid in rhENO1-treated samples (Fig. 6C). Notably, SM species SM (d43:7; O2) and SM (d19:1/20:4(5Z,8Z,11Z,14Z)-OH (20)) were markedly elevated (Fig. 6D). SM, a phospholipid critical for membrane structure and tumorigenic signaling, regulates proliferation, migration, and inflammatory responses [38, 39].

Sphingosine kinase 1 (SPHK1), a key enzyme in sphingolipid metabolism, catalyzes sphingosine-1-phosphate (S1P) biosynthesis. Growth factors and cytokines activate SPHK1 via ERK-dependent phosphorylation at Ser225, triggering its membrane translocation [40]. Given our findings linking ENO1 to SM metabolism, we investigated whether ENO1 activates SPHK1 through ERK signaling. Western blot analysis revealed that rhENO1 significantly enhanced phosphorylation of ERK and SPHK1 (Ser225) (Fig. 6E). Pretreatment with the TLR4 inhibitor TAK-242 abolished this effect (Fig. 6F), confirming TLR4-dependent regulation of the ERK/SPHK1 cascade. Immunofluorescence demonstrated rhENO1-induced SPHK1 membrane translocation, which was suppressed by TAK-242(10 μM) (Fig. 6G). Notably, the SPHK1 inhibitor PF-543 partially attenuated the pro-tumorigenic effects of rhENO1 on GBM cell proliferation, migration, and invasion (Supplementary Fig. 7A–D). These results establish a molecular mechanism whereby ENO1 activates the ERK/SPHK1/S1P axis via TLR4 to promote sphingolipid metabolism. Consistently, ELISA confirmed elevated S1P levels in conditioned medium from rhENO1-treated GBM cells (Fig. 6H). The SPHK1 inhibitor PF-543 [41] (a competitive sphingosine antagonist) dose-dependently suppressed SPHK1 phosphorylation (Fig. 6I). Functional assays demonstrated that PF-543 (10 μM) inhibited GBM proliferation, migration, and invasion, while exogenous S1P (5 μM) rescued these effects (Fig. 6J–M). Collectively, these findings demonstrate that soluble ENO1 activates the TLR4/ERK/SPHK1/S1P signaling cascade to drive GBM malignancy, thereby establishing an autocrine S1P loop that drives malignant progression in GBM, expanding our understanding of ENO1’s non-glycolytic roles and identifying novel therapeutic targets.

### Extracellular ENO1 and S1P synergistically promote M2-like polarization of tumor-associated macrophages

**Fig. 7:**
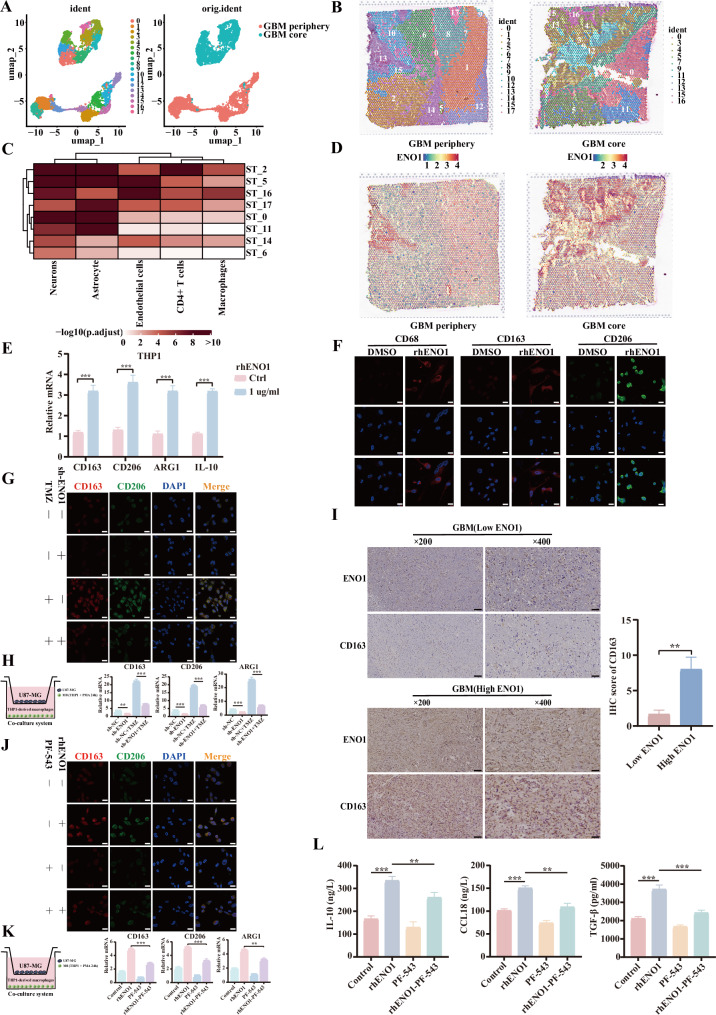
Extracellular ENO1 and S1P synergistically promote M2-like polarization of tumor-associated macrophages. **A** Dimensionality reduction and clustering of single-cell RNA sequencing (scRNA-seq) and spatial transcriptomic RNA sequencing (stRNA-seq) data from GEO database. **B** Cluster analysis of peritumoral and tumor core regions in GBM specimens. **C** MIA (Microenvironment Cell Populations) analysis evaluating cell type enrichment across different clusters in tumor core versus peritumoral regions. **D** Spatial distribution patterns of ENO1 expression in GBM tumor core and peritumoral regions. **E** qRT-PCR quantification of M2-TAM markers (CD163, CD206, ARG1, IL-10) mRNA levels following rhENO1 treatment (1 μg/mL). **F** Immunofluorescence analysis of M2-TAM markers (CD68, CD163, CD206) subcellular localization and expression intensity after rhENO1 treatment (1 μg/mL). Scale bar = 10 μm. **G** Immunofluorescence staining of M2-TAM markers (CD163, CD206) in TMZ-treated co-culture system of sh-ENO1 U87MG cells with M0 macrophages. Scale bar = 10 μm. **H** qRT-PCR analysis of M2-TAM markers (CD163, CD206, ARG1) mRNA expression in TMZ-treated sh-ENO1 U87MG/M0 macrophage co-culture system. **I** Immunohistochemistry (IHC) analysis of the correlation between CD163 and ENO1 in GBM tissues. **J** Confocal microscopy quantification of membrane localization intensity for M2-TAM surface markers (CD163/CD206) following treatment with rhENO1 (1 μg/mL) and SPHK1 inhibitor PF-543 (10 μM). Scale bar = 10 μm. **K** qRT-PCR detection of M2-TAM markers (CD163, CD206, ARG1) mRNA levels under combined rhENO1 (1 μg/mL) and PF-543 (10 μM) treatment. **L** The levels of cytokines IL-10, CCL18, and TGF-β released in the supernatant of THP-1-derived macrophages were detected by ELISA after indicated treatment for 24 h. Data are expressed as mean ± SEM. ns not significant, *P < 0.05, **P < 0.01, ***P < 0.001.

To explore the spatial heterogeneity of GBM and its cellular subpopulation distribution, we integrated single-cell RNA sequencing (scRNA-seq) and spatial transcriptomic RNA sequencing (stRNA-seq) datasets from the GEO database. After standardization of scRNA-seq data, PCA for dimensionality reduction, and analyses of cellular clustering and intercellular communication, we identified predominant secretory signaling pathways and complex regulatory networks among distinct cell subpopulations (Supplementary Fig. 8A–D). Notably, integrated analysis of scRNA-seq and stRNA-seq revealed a significant association between M2-macrophage accumulation and ENO1 level, as ENO1 expression was markedly upregulated in macrophage-enriched regions (Fig. 7A–D). This spatial co-localization implicates ENO1 as a key mediator of tumor cell-macrophage crosstalk.

Given the observed spatial association between ENO1 and macrophages in GBM, the role of soluble ENO1 was investigated in the polarization of TAMs. Accordingly, PMA (100 ng/mL)-differentiated M0 macrophages were treated with rhENO1 (1 μg/mL), resulting in significant upregulation of M2 markers (CD163, CD206, ARG1, and IL-10) via qRT-PCR (Fig. 7E). This finding was consistent with results of TCGA data analysis, showing positive correlations between ENO1 and M2-TAM markers (Supplementary Fig. 9A, B). IF staining confirmed elevated protein levels of CD68, CD163, and CD206 in rhENO1-treated macrophages (Fig. 7F). To assess whether TMZ-induced ENO1 secretion by GBM cells promotes M2 polarization via paracrine signaling, we established a novel co-culture system with PMA-induced THP-1-derived M0 macrophages in the lower chamber and ENO1-knockdown U87MG cells (shENO1) in the upper chamber. We found that shENO1 attenuated TMZ-induced upregulation of M2 markers (CD163, CD206, and ARG1) at both mRNA and protein levels (Fig. 7G, H), indicating that TMZ-induced M2 polarization depends on ENO1 expression in GBM cells. IHC analysis of human GBM specimens revealed a positive correlation between ENO1 expression and CD163^+^ M2-TAM infiltration in tumor stroma. High-power field (HPF) quantification showed significantly higher CD163^+^ cell density in ENO1-high specimens compared to ENO1-low specimens (Fig. 7I*P* < 0.01). These results collectively suggest that elevated ENO1 expression in GBM is associated with increased M2-TAM abundance in the tumor microenvironment.

To investigate the role of extracellular S1P in TAM phenotype remodeling, M0 macrophages were stimulated with S1P. The qRT-PCR analysis revealed dose-dependent upregulation of M2-TAM markers (CD163, CD206, ARG1, and IL-10) (Supplementary Fig. 10A), indicating S1P’s pro-M2 polarization function. Subsequent stimulation of U87MG cells with S1P significantly enhanced protein abundance and fluorescence intensity of CD68, CD163, and CD206 (Supplementary Fig. 10B). SPHK1 inhibitor PF-543 suppressed S1P secretion and reversed M2 polarization in a concentration-dependent manner (Supplementary Fig. 10C, D). In co-culture systems, PF-543 treatment significantly reduced expression of M2-TAMs markers (CD163, CD206, and ARG1) (Supplementary Fig. 10E). To eliminate potential confounding effects from direct cell-cell interactions, M0 macrophages were cultured in conditioned media from PF-543-treated U87MG cells. PF-543 reversed tumor-induced M2 polarization, as evidenced by decreased characteristic M2 marker expression (Supplementary Fig. 10F), confirming the role of soluble S1P in TAM reprogramming.

Combined treatment with rhENO1 and PF-543 revealed that SPHK1 inhibition reversed rhENO1-induced M2 polarization to some extent, as evidenced by reduced fluorescence intensity of CD163/CD206 and downregulated M2 marker mRNA levels (Fig. 7J, K). These findings establish that extracellular ENO1 and S1P work synergistically to drive M2 polarization of TAMs in GBM. Recent studies have demonstrated that M2-TAMs promote tumor progression through the secretion of diverse chemokines, growth factors, and cytokines [42]. THP-1 cells were stimulated with PMA to induce differentiation into M0 macrophages, which were then co-cultured with conditioned medium from tumor cells (Supplementary Fig. 11A). The results demonstrated that ENO1 overexpression significantly enhanced the recruitment of M0 macrophages, whereas ENO1 knockdown markedly attenuated this process (Supplementary Fig. 11B, C). Furthermore, ENO1 overexpression led to a significant increase in the secretion of IL-10, CCL18, and TGF-β in THP-1-derived macrophages, while ENO1 knockdown resulted in a notable reduction (Supplementary Fig. 11D, E). The levels of IL-10, CCL18, and TGF-β were significantly elevated in the supernatant of rhENO1-treated THP-1-derived macrophages. This effect was attenuated upon inhibition of S1P secretion using the SPHK1 inhibitor PF-543 (Fig. 7L). Collectively, these findings indicate that ENO1 promotes the secretion of IL-10, CCL18, and TGF-β in the TME by driving macrophage recruitment and polarization. This study reveals the pivotal role of ENO1 in regulating macrophage-mediated immunosuppression in GBM, potentially positioning it as a promising therapeutic target.

### Combined TMZ and TLR4/SPHK1 inhibition enhances anti-tumor efficacy

**Fig. 8:**
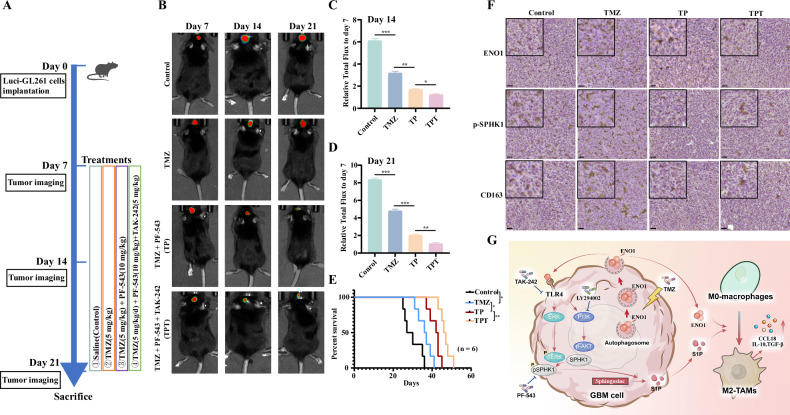
Combinatorial targeting of TLR4/SPHK1 enhances TMZ therapeutic efficacy in GBM. **A** Experimental workflow of orthotopic glioma mouse model and treatment regimen (n = 6). **B** Representative in vivo bioluminescence images of Luci-GL261-bearing mice treated with:(i) saline control, (ii) TMZ monotherapy, (iii) TMZ + PF-543 (SPHK1 inhibitor), (iv) TMZ + PF-543 + TAK242 (TLR4 inhibitor) at days 7, 14, and 21 post-implantation. **C**, **D** Quantitative analysis of tumor growth using relative flux values (normalized to day 7) across treatment groups. **E** Kaplan-Meier survival curves showing intracranial tumor progression in the four treatment cohorts; *n* = 6 mice. **F** Representative immunohistochemical staining of ENO1, p-SPHK1, and CD163 in tumor sections from each treatment group. Scale bar, 100 μm. **G** Schematic diagram depicting the mechanistic basis of ENO1 mediating cellular communication between GBM cells and M2-TAMs through an autophagy-dependent secretory pathway and driving TMZ chemoresistance. Data represent mean ± SEM; ns not significant, *P < 0.05, **P < 0.01, ***P < 0.001.

Building upon our previous findings that ENO1 mediates TMZ resistance in GBM, we innovatively evaluated a combinatorial therapeutic strategy targeting the SPHK1 inhibitor PF-543 and TLR4 antagonist TAK-242 alongside TMZ, leveraging its clinical pharmacokinetic profile (Fig. 8A). Intracranial tumor growth monitored by live imaging revealed that TMZ + PF-543 and TMZ + PF-543 + TAK-242 regimens significantly improved tumor suppression rates and prolonged the median survival of tumor-bearing mice compared to TMZ monotherapy. Notably, the triple combination (TMZ + PF-543 + TAK-242) yielded the highest anti-tumor efficacy among all treatment groups, including saline controls (Fig. 8B–E). IHC staining of post-treatment intracranial tumor sections quantified the expression of M2 macrophage markers ENO1, CD163, and phosphorylated SPHK1 (p-SPHK1). Histopathological scoring demonstrated significant downregulation of these biomarkers in TMZ + PF-543 and TMZ + PF-543 + TAK-242 treatment groups compared to monotherapy (Fig. 8F). Collectively, these results indicate that dual inhibition of TLR4-SPHK1 signaling synergizes with TMZ to enhance therapeutic efficacy in TMZ-resistant GBM, suppressing tumor growth and reversing M2-TAMs polarization in the tumor microenvironment.

## Discussion

GBM remains one of the most aggressive and therapeutically refractory malignancies, primarily due to its immunosuppressive TME, therapeutic resistance, and metabolic adaptability [43, 44]. In this study, we identified a novel mechanism by which autophagy-dependent secretion of ENO1 drives TMZ chemoresistance and immune evasion in GBM through bidirectional crosstalk between tumor cells and TAMs. We demonstrated for the first time that TMZ could activate secretory autophagy to promote extracellular release of ENO1, binding to TLR4 on GBM cells to trigger dual signaling cascades: PI3K/Akt activation and ERK/SPHK1/S1P signaling cascade. This axis not only enhanced GBM cell proliferation, migration, and invasion but also enabled soluble ENO1 and its downstream metabolite S1P to synergistically polarize TAMs toward an immunosuppressive M2 phenotype, thereby fostering a chemoresistant TME through pro-inflammatory chemokines and cytokines secretion. Critically, combinatorial targeting of TLR4 (TAK-242) and SPHK1 (PF-543) significantly enhanced TMZ efficacy, offering viable therapeutic approaches to overcome GBM chemoresistance (Fig. 8G).

Autophagy plays a dual role in cancer therapy, acting both as a pro-survival mechanism and as a mediator of cell death signals. While increased autophagy can promote therapeutic resistance by enabling cancer cell survival [45], secretory autophagy diverges from degradative pathways by facilitating unconventional protein secretion. Unlike proteins with N-terminal signal peptides secreted via the ER-Golgi pathway, proteins lacking canonical signal sequences rely on secretory autophagy for extracellular release [46–48]. For instance, 6-OHDA induces Park7 secretion via autophagy-based unconventional pathways in SH-SY5Y neuroblastoma cells and MEFs [49]. TMZ, the standard chemotherapeutic agent for GBM, activates autophagy with dual biological effects. While Li et al. reported that TMZ-induced secretory autophagy mediates HMGB1 release, which subsequently enhances TMZ sensitivity by polarizing TAMs toward an M1-like phenotype [50], the specific role of autophagy-dependent ENO1 secretion in remodeling the GBM TME remained unexplored. Our findings demonstrate that TMZ-induced ENO1 secretion requires autophagosome formation and depends on a functional autophagic flux. Pharmacological inhibition of autophagosome formation with 3-MA or impairment of autophagosome-lysosome fusion and subsequent degradation using BafA1 significantly reduced extracellular ENO1 levels, underscoring the essential role of TMZ-induced autophagy in promoting ENO1 release.

Growing evidence highlights secreted proteins as critical mediators of intercellular communication in the TME, influencing drug response and tumor progression [51]. ENO1, traditionally recognized as a glycolytic enzyme, exhibits non-canonical functions as a secreted protein. Tumor-derived secretory proteins, often overexpressed and aberrantly released, play pivotal roles in cell signaling, migration, and chemoresistance. While prior studies localized ENO1 to the cytoplasm and cell membrane, its extracellular secretion under stress conditions positions it as a prognostic biomarker and therapeutic target [52, 53]. Quantitative proteomics comparing ATG5-proficient and ATG5-deficient macrophages revealed reduced ENO1 secretion in autophagy-impaired cells [31]. While autophagy is widely recognized as a cytoprotective mechanism in cancer therapy, its role in mediating secretory processes—particularly in the context of chemoresistance—remains underexplored. Besides, Martinelli et al. identified ENO1 as a dexamethasone-induced secretory autophagy cargo via LC-MS/MS, dependent on ATG5-mediated autophagy [34]. Building on these findings, we demonstrated that TMZ could enhance autophagy-dependent ENO1 secretion in GBM via a non-canonical pathway independent of conventional ER-Golgi trafficking. This process, resembling IL-1β secretion [31], involves lysosomal damage and dynamic autophagic flux, suggesting that extracellular ENO1 remodels the TME through paracrine signaling to drive metabolic reprogramming in neighboring tumor or immune cells, thereby contributing to GBM malignancy, fostering immune evasion, and potentially mediating resistance to TMZ.

Our findings demonstrated that autophagy-dependent ENO1 secretion significantly enhanced GBM proliferation, migration, and invasion. This aligned with the findings of Jiang et al., who reported exosomal ENO1-mediated intercellular crosstalk in hepatocellular carcinoma via the integrin α6β4/FAK/Src-p38-MAPK axis [28]. Notably, the administration of exogenous recombinant ENO1 (rhENO1) effectively mitigated the proliferative and invasive deficits in ENO1-knockdown GBM cells, indicating extracellular activity independent of intracellular glycolytic functions, likely mediated by autocrine/paracrine signaling via cell-surface receptors. Survival analyses conducted on TCGA and CGGA datasets revealed that high ENO1 expression correlated with poor prognosis in GBM patients and was significantly upregulated in recurrent gliomas, suggesting its potential as an independent prognostic biomarker. Notably, TMZ treatment elevated serum ENO1 levels, likely due to autophagy-driven unconventional secretion, establishing a “therapy-secretion-resistance” feedback loop. This underscored the role of the “autophagy-secretion-TME remodeling” axis in chemoresistance and suggested that extracellular ENO1 accumulation exacerbated therapeutic resistance by recruiting immunosuppressive cells, offering a novel mechanistic explanation for GBM treatment failure.

Our investigation further identified a direct interaction between ENO1 and TLR4 on GBM cells mediated by hydrogen bonding, consistent with prior reports of ENO1-TLR4 signaling in immune activation [27]. Importantly, the present study established its functional significance in GBM. Notably, the TLR4 inhibitor TAK-242 reversed ENO1-induced malignant phenotypes, demonstrating that TLR4 is indispensable for ENO1 signaling and acts as a core mediator downstream of ENO1. This discovery provides novel insights into how secretory proteins regulate tumor cell behavior through membrane receptors. Transcriptomic profiling of rhENO1-stimulated GBM cells revealed significant enrichment of PI3K/Akt signaling pathways. Functional validation showed that PI3K inhibitor LY294002 partially attenuated rhENO1-driven proliferation, migration, and invasion, suggesting co-activation of alternative pro-tumorigenic pathways beyond PI3K/Akt. Beyond its glycolytic function, ENO1’s moonlighting activities have recently attracted significant interest. Untargeted lipidomics revealed ENO1’s involvement in SM metabolism, with SM accumulation driving GBM malignancy. Dysregulated sphingolipid metabolism, increasingly recognized as a chemoresistance mechanism, was exemplified by Rao et al. [54], who demonstrated that SETD2 loss enhances SM biosynthesis, promoting renal cancer progression. Current evidence suggests that tumor cells overexpress sphingosine kinase 1 (SPHK1), a pro-tumorigenic enzyme responsible for synthesizing sphingosine-1-phosphate (S1P), a key signaling molecule in the TME implicated in proliferation, angiogenesis, metastasis, chemoresistance, metabolic reprogramming, and immune escape [55, 56]. Our study mechanistically linked ENO1-TLR4 signaling to SPHK1 activation and S1P production, with TLR4/SPHK1 inhibitors (TAK-242/PF-543) blocking this axis. These findings position the ENO1/TLR4-SPHK1/S1P axis as a metabolic-immune axis in GBM, where S1P sustains malignancy through autocrine signaling, expanding ENO1’s moonlighting functions and highlighting its therapeutic potential.

It is now understood that bidirectional crosstalk between cells and their microenvironment is critical for tumor progression [57]. Interactions between tumor cells and macrophages play a pivotal role in cancer development, supporting angiogenesis, nurturing cancer stem cells, and fostering an immunosuppressive TME. GBM is characterized by a “cold” tumor with abundant TAMs infiltration. These TAMs typically exhibit an M2-like phenotype and promote angiogenesis, immune suppression, and therapy resistance [58, 59]. Beyond direct cell-cell contact, paracrine signaling via cytokines, chemokines, and metabolites governs TME communication. For instance, β2-microglobulin (B2M) activates SMAD and PI3K/AKT/mTOR pathways in TAMs to drive M2 polarization [60]. Besides, a recent study showed that glioma-derived kynurenine activates the aryl hydrocarbon receptor (AHR) in TAMs, modulating their recruitment and inducing T-cell dysfunction [61], while glioma stem cell-derived POSTN recruits TAMs [62]. Our study reveals a novel mechanism by which ENO1 shapes the tumor immune microenvironment (TIME) in GBM through promoting macrophage polarization. Mechanistic studies of the TME regulation reveal that ENO1 and sphingosine-1-phosphate (S1P) act as critical mediators driving TAMs polarization toward an M2 phenotype through their interaction network with malignant cells, thereby promoting immune evasion and chemoresistance. Our study systematically revealed how extracellular ENO1 and S1P synergize to polarize TAMs toward an M2 phenotype, thereby reshaping the immunosuppressive TME. Integrated analysis of scRNA-seq and stRNA-seq revealed spatial co-localization of *ENO1*-high regions with TAM-enriched zones, providing histological evidence that ENO1 overexpression in GBM tissues is correlated with increased M2-polarized TAMs infiltration within tumor stroma. Functionally, both rhENO1 and S1P independently upregulated M2 markers (CD163/CD206/ARG1/IL-10), while PF-543 attenuated these effects, underscoring their synergistic regulation. Notably, TMZ-induced ENO1 secretion exacerbated S1P-dependent M2 polarization, leading to the release of chemokines and cytokines including CCL18, IL-10, and TGF-β, offering a novel explanation for chemoresistance. Combined inhibition of TLR4 and SPHK1 synergizes with TMZ to suppress tumor growth and reverse M2 macrophage polarization in vivo, highlighting the therapeutic promise of targeting the ENO1/TLR4-SPHK1 axis.

Despite these significant findings, there are some limitations in our study. First, although we confirmed the ENO1-TLR4 interaction in GBM, the precise binding sites remain undefined and require further investigation. Second, the detailed mechanisms underlying ENO1-mediated macrophage polarization in GBM has yet to be fully elucidated. Subsequent studies should delve into the signaling cascades mediated by ENO1 that drive M2-TAM polarization, elucidate how these pathways interact with other immune and stromal components within the TIME, and further investigate the cross-regulatory mechanisms between GBM and the TIME to enhance therapeutic efficacy. Additionally, future research utilizing organoid models holds promise for elucidating the role of ENO1 in GBM progression and drug resistance mechanisms.

Collectively, our findings demonstrate that TMZ-induced autophagy-dependent ENO1 secretion activates two pivotal signaling axes, TLR4-mediated PI3K/Akt and SPHK1/S1P pathways, to drive glioblastoma malignancy. Secreted ENO1 and S1P synergistically drive M2-like TAM polarization via paracrine signaling, fostering an immunosuppressive TME that underpins chemoresistance. Our findings elucidate the central role of secretory autophagy in TME remodeling and validate the therapeutic potential of combining TLR4/SPHK1 inhibitors with TMZ to enhance efficacy and reverse resistance. This work deepens our understanding of tumor-immune crosstalk and provides a rationale for the development of precision therapies targeting metabolic-immune interplay in GBM.

## Materials and methods

### Cell line and culture

Human glioblastoma cell lines (U87MG, T98G, U118, A172, LN229, U251) were obtained from the American Type Culture Collection (ATCC). The human monocytic leukemia cell line (THP-1) was purchased from the Cell Bank/Stem Cell Bank of the Chinese Academy of Sciences. Cell authentication was confirmed by short tandem repeat analysis. GBM cells were maintained in DMEM high-glucose medium supplemented with 10% fetal bovine serum (FBS), while THP-1 cells were cultured in complete RPMI-1640 medium. All cell lines were incubated at 37 °C in a humidified atmosphere containing 5% CO₂, with medium refreshed every 48 h. Routine testing confirmed all cell lines were free of mycoplasma contamination.

### Bioinformatics and Statistical Analysis

We systematically analyzed open-access datasets from TCGA (https://portal.gdc.cancer.gov/) and CGGA (http://www.cgga.org.cn/), incorporating whole transcriptome RNA sequencing (RNA-Seq) data from low-grade gliomas (WHO grades II-III) and glioblastomas (WHO grade IV). Multidimensional clinicopathological parameters were collected, including demographic characteristics (age, sex); disease progression markers (WHO grade, pathological subtype); molecular classification (IDH mutation status, 1p/19q co-deletion status), and survival outcomes. Kaplan-Meier survival analysis was performed to evaluate the association between ENO1 expression and overall survival (OS), with corresponding survival curves generated.

For multimodal integration analysis, we utilized single-cell RNA sequencing (scRNA-seq; GSE273274) and spatial transcriptomic RNA sequencing (stRNA-seq; GSE273275) data from the GEO database. Both datasets underwent PCA, dimensionality reduction and cell clustering analysis. Multimodal Intersection Analysis (MIA) was employed to identify spatially enriched cell types within specific tissue regions or cellular clusters.

### Antibodies

For Western blot analysis, primary antibodies were diluted at 1:1000 to 1:2000, with corresponding secondary antibodies used at 1:5000. For both immunohistochemical (IHC) staining and immunofluorescence (IF) assays, primary antibodies were diluted at 1:50 to 1:400. The following antibodies were employed in both Western blot and IHC experiments: ENO1 (Proteintech, 11204-1-AP), TLR4 (Abcam, ab22048), SEC22B (Proteintech, 14776-1-AP), TRIM16 (Proteintech, 24403-1-AP), Akt (Immunoway, YM8463), p-Akt (Immunoway, YM8531), PI3K (Cell Signaling Technology, 4257), p-PI3K (Cell Signaling Technology, 17366), CD163 (Abcam, ab156769), CD206 (Proteintech, 18704-1-AP), SPHK1 (Proteintech, 10670-1-AP), p-SPHK1 Ser225 (Proteintech, 19561-1-AP), ERK (Cell Signaling Technology, 4695), p-ERK (Cell Signaling Technology, 4370), FN1 (Proteintech, 15613-1-AP), GAL-3 (Proteintech, 14979-1-AP), GAL-8 (Abcam, ab109519), GAPDH (Proteintech, 10494-1-AP), anti-Rabbit HRP-conjugated secondary antibody (Cell Signaling Technology,7074), anti-Mouse HRP-conjugated secondary antibody (Cell Signaling Technology,7076).

### Reagents

Recombinant protein ENO1 (Cat# HY-P70260A) was purchased from MedChemExpress (MCE). The chemical reagents used in this study included: temozolomide (TMZ, MedChemExpress, HY-17364), dimethyl sulfoxide (DMSO, MedChemExpress, HY-Y0320), bafilomycin A1 (Baf-A1, Selleck, S1413), brefeldin (BFA, Selleck, S7046), 3-Methyladenine (3-MA, MedChemExpress, HY-19312), TAK-242 (Selleck, S7455), LY294002 (Selleck, S1105), PF-543 (MedChemExpress, HY-15425), Sphingosine-1-Phosphate (S1P, MedChemExpress, HY-108496).

### Western blot analysis

Cells were lysed in RIPA buffer (Fdbio Science, Hangzhou, China) supplemented with protease and phosphatase inhibitors. Protein concentrations were determined using a BCA protein assay kit (Beyotime, Shanghai, China). Protein samples were separated by SDS-PAGE and subsequently transferred to PVDF membranes (Millipore, IPVH00010, USA) at a constant current of 320 mA for 2 h. The membranes were then blocked with 5% non-fat milk (BD Biosciences, 232100, USA) in TBST for 1–2 h at room temperature, followed by incubation with primary antibodies at 4 °C overnight. After washing three times with TBST (10 min per wash), the membranes were incubated with HRP-conjugated secondary antibodies (anti-rabbit or anti-mouse) for 1–2 h at room temperature. Protein bands were visualized using an ECL detection kit (Biosharp, BL520B, China) under dark conditions.

### Co‑immunoprecipitation (Co‑IP) assay

Cells were washed three times with pre-chilled PBS and lysed using IP lysis/wash buffer supplemented with protease and phosphatase inhibitors. Cell lysates were incubated overnight at 4 °C with Protein A/G magnetic beads pre-conjugated with target-specific antibodies. The immunocomplexes were washed three times with 200 μL of pre-chilled IP lysis buffer using magnetic separation. After removing the supernatant, the beads were resuspended in 1× SDS-PAGE loading buffer, heat-denatured, and briefly centrifuged for 10 s. The protein-containing supernatant was collected by magnetic separation for subsequent Western blot analysis. Antibodies used for target protein IP included: ENO1 Rabbit pAb (Proteintech, Cat# 11204-1-AP; 1:100 dilution), SEC22B Rabbit pAb (Proteintech, Cat# 14776-1-AP; 1:100 dilution), TRIM16 Rabbit pAb (Proteintech, Cat# 24403-1-AP; 1:100 dilution), TLR4 Mouse mAb (Abcam, Cat# ab22048; 1:100 dilution) and control IgG (Proteintech, Cat# 30000-0-AP; 1:100 dilution).

### Quantitative real-time PCR (RT-qPCR)

Total RNA was extracted using TRIzol reagent (Invitrogen, 15596-026, USA), with concentration and purity assessed by measuring absorbance at 260/280 nm using a NanoDrop spectrophotometer. RNA was subsequently reverse transcribed into cDNA using a Reverse Transcription Kit (Thermo Fisher, K1622, USA). Quantitative real-time PCR (qPCR) was performed on a QuantStudio 6 Real-Time PCR System using 2× ChamQ Blue Universal SYBR qPCR Master Mix (Vazyme, Nanjing, China). GAPDH served as the endogenous control for cDNA normalization. All reactions were performed in triplicate technical replicates. Complete primer sequences are provided in Supplementary Table 1.

### Conditioned medium(CM) preparation and analysis

Cells were initially cultured in complete medium supplemented with 10% fetal bovine serum for 24–48 h, followed by three washes with PBS. The medium was then replaced with serum-free conditioned medium (SFCM) for an additional 24–48 h incubation period. The collected supernatant underwent sequential processing, beginning with an initial centrifugation at 1000 × *g* for 10 min to remove cellular debris, followed by concentration using 10 kDa molecular weight cutoff ultrafiltration tubes (Millipore, UFC901096) at 5000 × *g* for 50 min. Total protein concentrations in both the concentrated SFCM and whole cell lysates (WCL) were quantified by BCA assay. Western blot analysis was performed to detect target proteins in SFCM and WCL samples.

### Enzyme-Linked Immunosorbent Assay (ELISA)

The concentrations of ENO1 and sphingosine-1-phosphate (S1P) in GBM cell culture supernatants and patient serum samples were determined using commercial ELISA kits according to the manufacturers’ protocols: human ENO1 ELISA Kit (Abcam, #ab181417) and human S1P ELISA Kit (LabRe, #LB9693A). The concentrations of IL-10 (Cat# MM-0066H2), CCL18 (Cat# MM-1774H2), and TGF-β (Cat# MM-60157H2) in the supernatant of co-cultured GBM and THP-1-derived macrophages were quantified using ELISA, from MEIMIAN. Protein concentrations were calculated by extrapolating absorbance values against standard curves generated for each assay. All measurements were performed in technical duplicates/triplicates.

### Molecular docking analysis and visualization of ENO1-TLR4 interaction

The crystal structures of ENO1 (PDB ID: 2PSN) and TLR4 (PDB ID: 8WO1) were retrieved from the RCSB Protein Data Bank (https://www.rcsb.org/). Protein structures were further validated using AlphaFold predictions (https://alphafold.com). Prior to docking simulations, both structures were processed using AutoDockTools-1.5.7 to remove water molecules and small compounds and optimize hydrogen bonding networks. Molecular docking was performed with PyMOL software, with ENO1 designated as the ligand and TLR4 as the receptor. The resulting complexes were visualized and analyzed for potential interaction interfaces.

### Transmission electron microscopy (TEM)

Following pretreatment with either 600 μM TMZ or DMSO vehicle control, cells were washed twice with pre-colded PBS and collected in sterile microcentrifuge tubes. Samples were fixed overnight at 4 °C by gentle addition of pre-chilled 2.5% glutaraldehyde solution along the tube walls. Fixed specimens underwent sequential dehydration through an ethanol gradient series (70–100%) followed by acetone treatment. For TEM analysis, samples were processed by a specialized electron microscopy core facility, where they were embedded, sectioned (70 nm ultrathin sections), and stained with uranyl acetate and lead citrate. TEM imaging was performed using a JEOL JEM-1400 Flash transmission electron microscope (JEOL, USA).

### Cell viability assay

Cells were seeded in 96-well plates at a density of 2000 cells/well, with six biological replicates per treatment group plus blank control wells. Plates were incubated at 37 °C with 5% CO₂ for 24 h to allow cell attachment. Following CCK-8 reagent treatment, cells were incubated with the working solution for precisely 1 h at standard culture conditions. Cellular viability was quantitatively assessed through daily absorbance measurements at 450 nm for 7 consecutive days using a microplate reader. The mean optical density (OD) values from biological replicates were used for final data analysis.

### Cell colony formation assay

To evaluate proliferative capacity, cells were seeded in 6-well plates at a density of 1000 cells/well in complete medium supplemented with 10% FBS. Following cell attachment (typically 24 h), treatment regimens were administered according to experimental groups. Following treatment, cells were maintained in a 37 °C, 5% CO₂ humidified incubator for 10–14 days to allow colony formation, with medium refreshed every 3 days. The cells were fixed with 4% paraformaldehyde, and the colonies were stained and counted using 1% crystal violet. For each experimental group, three independent assays were performed.

### EdU proliferation assay

Cell proliferation was assessed using an EdU detection kit (RIBOBIO; C10310-1) according to the manufacturer’s protocol. Briefly, GBM cells were seeded on coverslips in 6-well plates and cultured for 24 h in a humidified incubator at 37 °C with 5% CO₂. The cells were then pulsed with 25 μM EdU for 2 h, fixed with 4% paraformaldehyde, and permeabilized with 0.5% Triton X-100 for 10 min. Afterward, Apollo fluorescent dye was applied for 30 min in the dark. Images were acquired using a fluorescence microscope.

### Wound healing assay

Cell migration was assessed using a scratch wound healing assay. Briefly, serum-starved U87MG or LN229 cells were seeded into 6-well plates at an appropriate density and cultured for 24 h. A straight wound was created in the monolayer using a 200 μL pipette tip, followed by three PBS washes to remove detached cells. The cells were then maintained in serum-free medium at 37 °C with 5% CO₂. Wound closure was monitored at 0, 24, and 48 h using an inverted phase-contrast microscope, and the migrated area was quantified with ImageJ software.

### Transwell migration and invasion assays

GBM cells suspended in serum-free medium were seeded into the upper chamber of Transwell inserts (8 μm pore size; Corning, USA) at a density of 3 × 10⁴ cells per well, with or without Matrigel coating (Corning, USA) for invasion and migration assays, respectively. The lower chamber was filled with 600–800 μL of chemoattractant medium containing 15% FBS. After incubation for 12–48 h at 37 °C with 5% CO₂ (duration depending on cell migratory capacity), cells were fixed with 4% paraformaldehyde at room temperature for 30 min and stained with 0.1% crystal violet for 10–20 min. Five random fields per insert were imaged using an inverted microscope (×200 magnification). Migrated/invaded cells were counted using ImageJ software, with data from three independent experiments averaged for statistical analysis.

### Macrophage-GBM coculture system

THP1 cells were first seeded in the lower chambers (Corning, USA) and differentiated into M0-type TAMs by treatment with 100 ng/mL phorbol 12-myristate 13-acetate (PMA). Concurrently, U87MG cells were seeded in the upper chambers (0.4 μm pore size; Corning, USA) at a density of 2 × 10⁵ cells/mL. After 24 h of separate culture, the macrophage-loaded lower chambers were assembled with GBM cell-loaded upper chambers. Following 24–72 h of coculture under various treatment conditions, either cells or conditioned media were collected for subsequent experiments according to the study design.

### Immunohistochemistry staining and evaluation

Formalin-fixed, paraffin-embedded tissue sections were deparaffinized and rehydrated, followed by antigen retrieval using 0.01 M citrate buffer (pH 6.0). Endogenous peroxidase activity was quenched with 3% hydrogen peroxide. Sections were then incubated overnight at 4 °C with primary antibodies against ENO1 (1:500), p-SPHK1 (1:200), p-ERK (1:100), and LC3B (1:100). After washing, sections were treated with HRP-conjugated secondary antibody (1:1000, A21020, Abbkine, Wuhan) for 1 h at room temperature. Color development was achieved using DAB substrate, followed by counterstaining with hematoxylin. Semi-quantitative analysis was conducted by multiplying the staining intensity score by the percentage of positive cells. Staining intensity was graded as: 0 (negative), 1 (weak, light yellow), 2 (moderate, yellowish brown), or 3 (strong, dark brown). The percentage of positive cells was scored as: 0 (0%), 1 (1–25%), 2 (26–50%), 3 (51–75%), or 4 (76–100%). Images were acquired using a light microscope.

### Immunofluorescence (IF) Staining

Cells were fixed with 4% paraformaldehyde for 10 min and permeabilized with 0.3% Triton X-100 for 10 min at room temperature. After blocking with 5% BSA for 1 h, cells were incubated overnight at 4 °C with primary antibodies diluted in 5% BSA. The following day, cells were stained for 1 h at room temperature (protected from light) with Alexa Fluor 488- (1:100, Invitrogen, A10436) or Alexa Fluor 555-conjugated secondary antibodies (1:100, Invitrogen, A20501MP). Nuclei were counterstained with DAPI for 5 min in the dark. Images were acquired using a confocal microscope and analyzed with ZEN 3.2 software.

### Construction of stable cells

For viral transduction, cells were seeded at a density of 5 × 10⁵ cells/well in culture dishes to achieve 30-40% confluence, followed by overnight incubation at 37 °C with 5% CO₂. Viral particles in the culture medium were quantitatively added to the system. After 12–20 h of infection, cell viability was assessed, and the medium was replaced within 24 h post-transduction. Selection with 5 μg/ml puromycin-containing medium was initiated at 72 h post-infection to establish stable pools, with regular passaging for expansion. Transduction efficiency was evaluated by Western blot analysis or RT-qPCR. The shRNA sequences used are provided in Supplementary Table S2.

### RNA Sequencing (RNA-Seq)

Total RNA was extracted from control and rhENO1-stimulated GBM cells using TRIzol reagent (Invitrogen, 15596-026) following the manufacturer’s protocol. RNA quality was assessed using Nanodrop 2000 (Thermo Fisher) and Bioanalyzer 2100 (Agilent). Libraries were prepared from high-quality RNA samples and sequenced on an Illumina Novaseq 6000 platform (150 bp paired-end reads). Differential gene expression analysis was performed using edgeR, with KEGG pathway enrichment analysis applied to identify significantly enriched functional pathways among differentially expressed genes (DEGs).

### Untargeted lipid metabolomics analysis

Untargeted lipid metabolomics analysis was performed on rhENO1-treated specimens. All chromatographic separations were carried out using an ACQUITY UPLC system (Waters, Milford, MA, USA). Reverse-phase separation was achieved with a Kinetex UPLC C18 column (100 mm × 2.1 mm, 100 Å; Phenomenex, UK). Metabolites eluted from the column were detected using a high-resolution tandem mass spectrometer TripleTOF 6600 (SCIEX, Framingham, MA, USA) and a Q-Exactive HF-X mass spectrometer (Thermo Scientific, Waltham, MA, USA). Raw mass spectrometry data were preprocessed with XCMS software for peak picking, peak alignment, secondary alignment, and isotope/adduct annotation. Statistical analyses were conducted using R software (version 4.1.0).

### Xenograft models

GL261 cells (1 × 10⁶ cells in 5 μL PBS) were stereotactically implanted into the frontal cortex (coordinates: 2 mm lateral, 4 mm posterior, and 3.5 mm ventral to bregma) of 5-week-old female C57BL/6 mice. Mice were allocated randomly into four groups (n = 6/group). Tumor formation was confirmed by in vivo fluorescence imaging (ImageViewer) at 7 days post-implantation. Beginning on day 7 post-surgery, mice received daily treatments with: temozolomide (5 mg/kg/day), TAK-242 (5 mg/kg/day), PF-543 (10 mg/kg/day), LY294002(5 mg/kg/day), and vehicle control (equal volume of saline). Tumor progression was monitored by bioluminescent imaging on days 7, 14, and 21. Following terminal anesthesia, mice underwent transcardial perfusion with heparinized saline followed by 4% paraformaldehyde (PFA) for brain extraction. Tumor volume was calculated using the formula: V = (length × width × height) × 0.5.

### Statistical analysis

All experiments were independently repeated at least three times. All statistical analyses were performed using R software (version 4.1.0) and GraphPad Prism (version 10.1.0). Data are presented as mean ± standard deviation (SD). Comparisons between two groups were analyzed using unpaired Student’s t-test, while the Mann-Whitney U test was applied for non-normally distributed data. One-way analysis of variance (ANOVA) was used for multiple group comparisons. A P < 0.05 was considered statistically significant, with significance levels denoted as *P < 0.05, **P < 0.01, and ***P < 0.001.

## Supplementary information

The full length uncropped original western blots

Supplementary information

Supplementary Figure 1

Supplementary Figure 2

Supplementary Figure 3

Supplementary Figure 4

Supplementary Figure 5

Supplementary Figure 6

Supplementary Figure 7

Supplementary Figure 8

Supplementary Figure 9

Supplementary Figure 10

Supplementary Figure 11

Supplementary Table

## Data Availability

The data that support the findings of this study are available from the corresponding author upon reasonable request. All data generated or analyzed in this study are included in the article and its supplementary files.
