# Supplementary material for: Autophagy-dependent secretion of ENO1 mediates chemoresistance of glioblastoma and tumor microenvironment remodeling
Source: Cell Death Dis. 2025 Dec 6;17(1):79. doi: 10.1038/s41419-025-08313-5 (PMC12827997; doi:10.1038/s41419-025-08313-5)

Autophagy-dependent secretion of ENO1 mediates chemoresistance of glioblastoma and tumor microenvironment remodeling

Qijun Xie[1,2,3,4†], Lei Chen[1,2,3,4†], Yifeng Huang[1,2,3,4†], Ziyuan Yu[1,2,3,4†], Rongzhang Zhu[1,2,3,4†], Junjie Li[1,2,3], Jiakun Zhao[1,2,3], Yiqi Song [1,2,3], Hong Li[1,2,3], Yuntao Lu[1,2,3]*

uncropped western blots

Figure. 1D

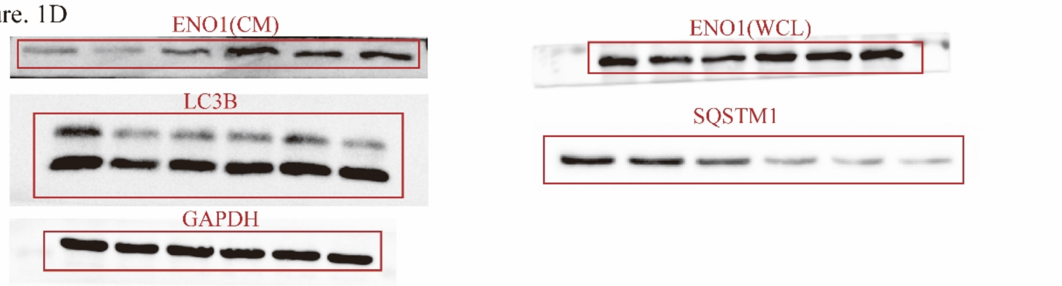

Figure. 1E

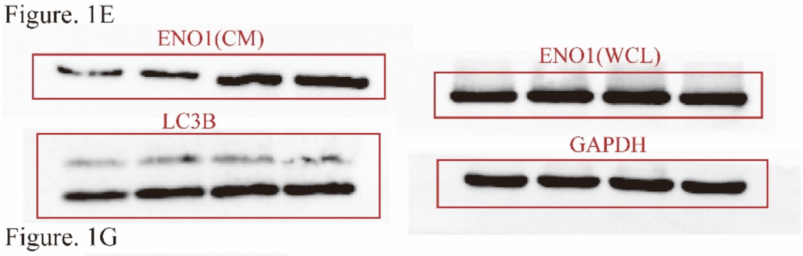

Figure. 1G

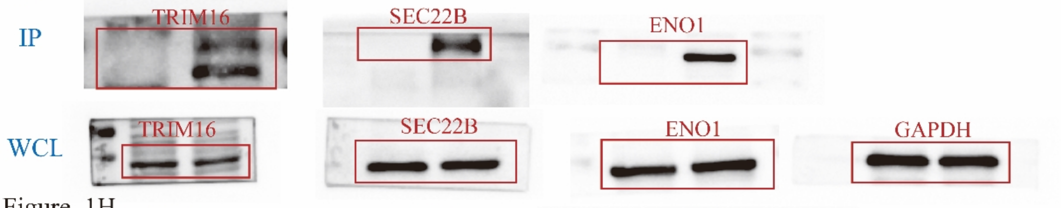

Figure. 1H

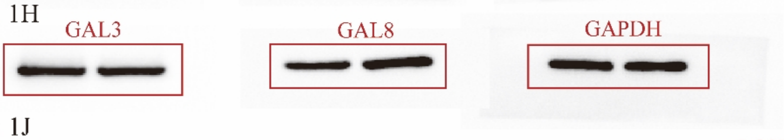

Figure. 1J

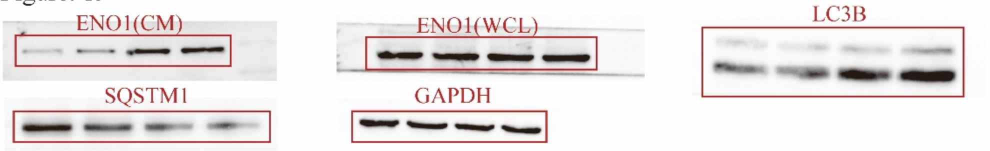

Figure. 1K

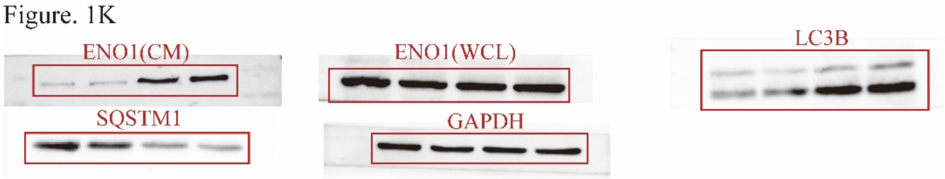

Figure. 1L

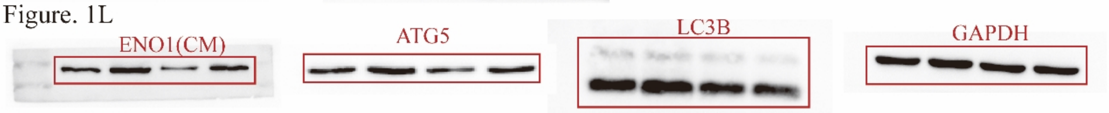

Figure. 1M

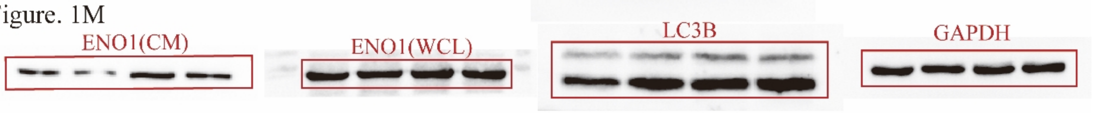

Figure. 1N

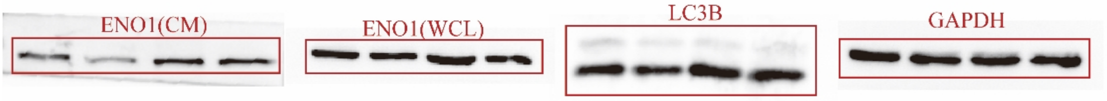

Figure. 3A

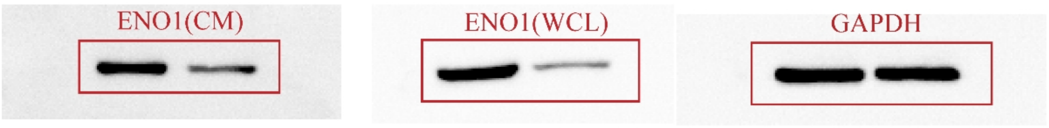

Figure. 3B

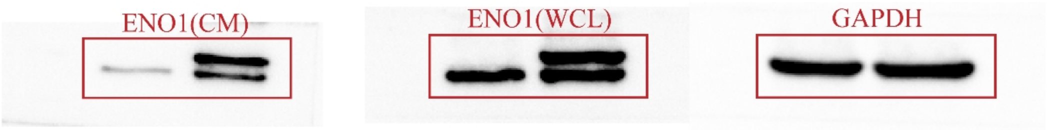

Figure. 3G

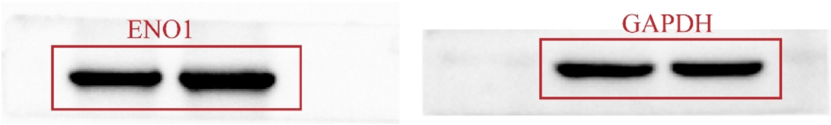

Figure. 4D

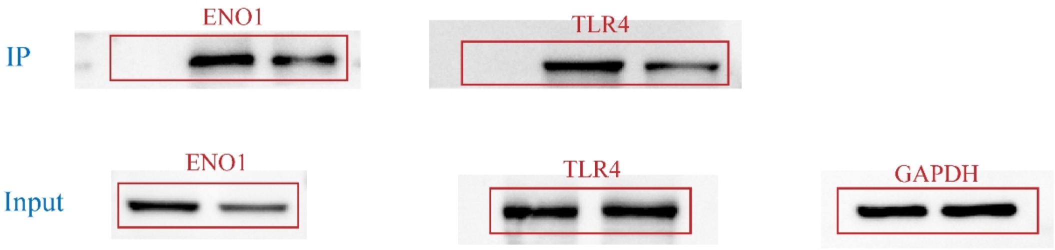

Figure. 4E

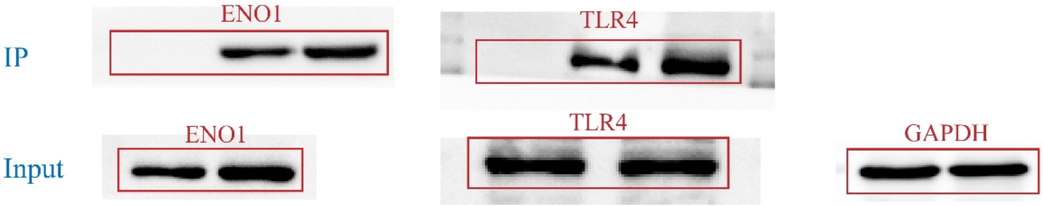

Figure. 4F

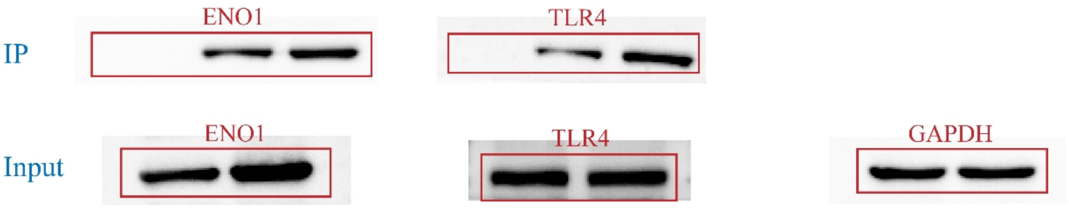

Figure. 5E

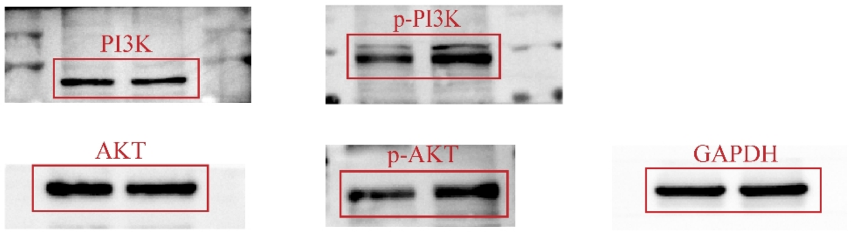

Figure. 5F

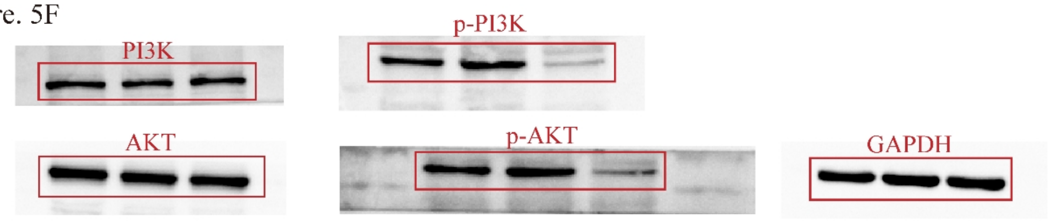

Figure. 5K

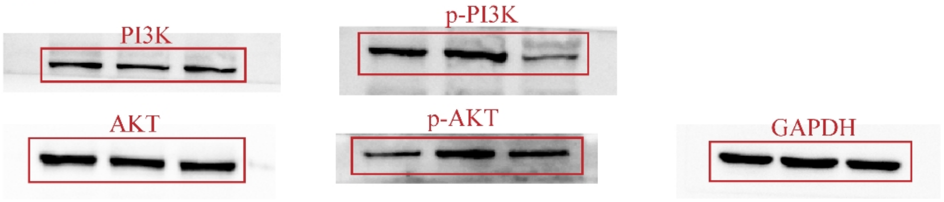

Figure. 6E

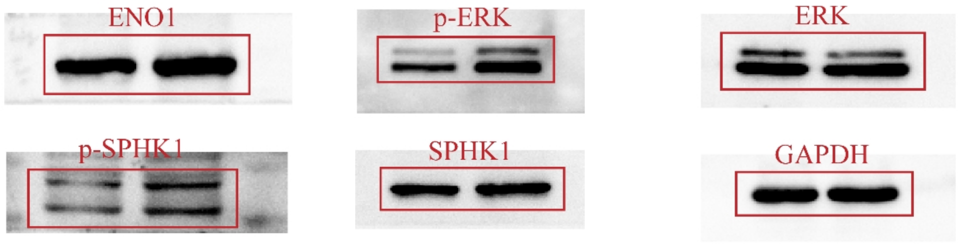

Figure. 6F

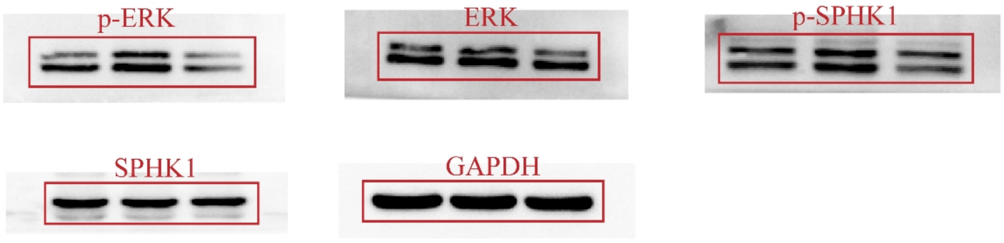

Figure. 6I

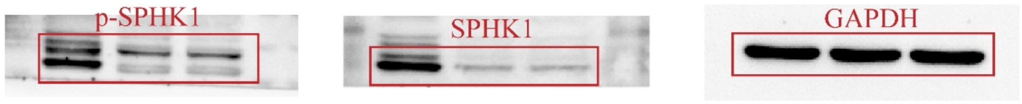

Figure. S1B

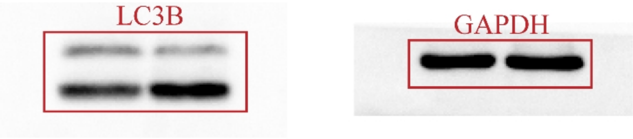

Figure. S1D

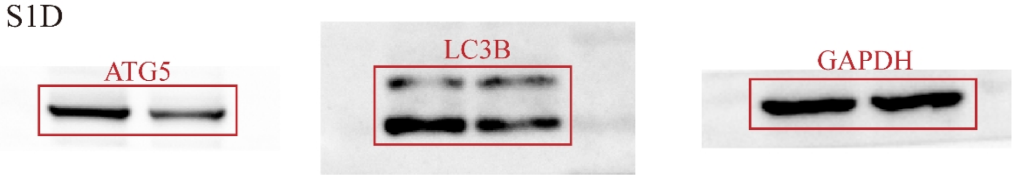

Figure. S2A

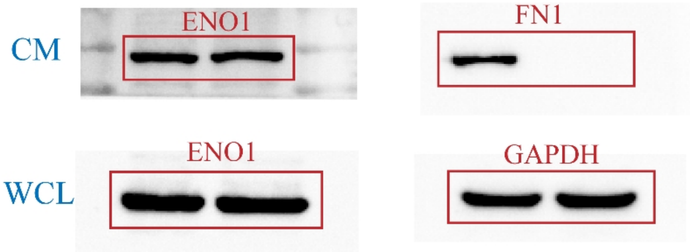

Figure. S3H

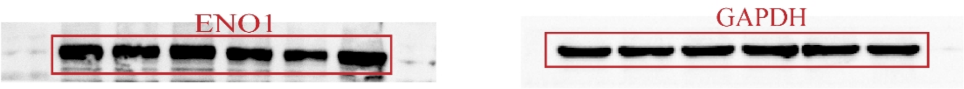

Figure. S3I

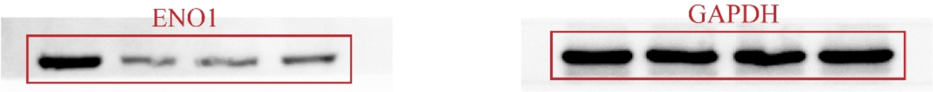

Figure. S3J

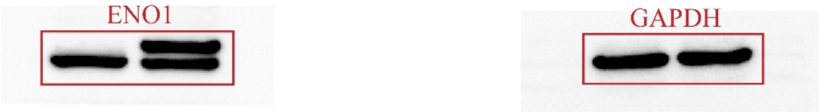

Figure. S4A

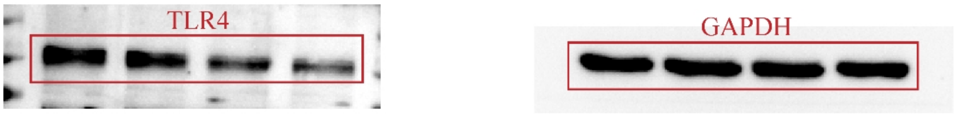

Figure. S4B

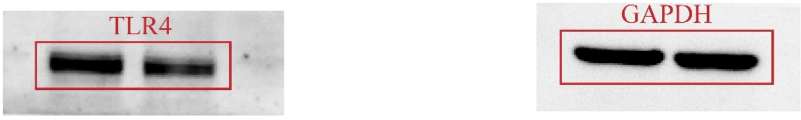

Supplement: Supplementary file 1 — The full length uncropped original western blots [file 41419_2025_8313_MOESM1_ESM.pdf]
