# Supplementary material for: Autophagy-dependent secretion of ENO1 mediates chemoresistance of glioblastoma and tumor microenvironment remodeling
Source: Cell Death Dis. 2025 Dec 6;17(1):79. doi: 10.1038/s41419-025-08313-5 (PMC12827997; doi:10.1038/s41419-025-08313-5)
Supplement: Supplementary file 2 — Supplementary information [file 41419_2025_8313_MOESM2_ESM.docx]

**Supplementary Figure Legends**

**Supplementary Figure 1 TMZ-induced autophagy-derived factors modulate GBM biology**. **A** Immunofluorescence staining of LC3B-positive puncta in GBM cells treated with TMZ (600 μM, 48 h). Scale bars, 20 μm. **B** Western blot analysis of LC3B-I to LC3B-II conversion in GBM cells following TMZ treatment (600 μM, 48 h). **C** Transmission electron microscopy (TEM) images demonstrating autophagosome formation in TMZ-treated GBM cells (600 μM, 48 h). Scale bars, 500 nm. The red arrow points to the double membrane autophagosome-like vesicle. **D** Western blot validation of ATG5-knockdown efficiency via ATG5 and LC3B expression in shATG5 cells. **(E, F)** EDU incorporation assay **(E)** and wound healing assay **(F)** showing the stimulatory effects of conditioned medium from TMZ-treated shATG5 cells (600 μM, 48 h) on GBM cell proliferation and migration. Scale bars = 100 μm **(E)** and 500 μm **(F)**. Data are expressed as mean ± SEM. ns not significant, *P < 0.05, **P < 0.01, ***P < 0.001.

**Supplementary Figure 2 ENO1 is secreted via unconventional secretory pathways in GBM cells. A** Western blot analysis of indicated proteins in whole-cell lysates (WCL) and conditioned media (CM) from GBM cells treated with or without Brefeldin A (BFA, 2 μg/ml) under serum-free conditions for 12 h. **B** Signal peptide prediction for ENO1 using SignalP-6.0 (<https://services.healthtech.dtu.dk/services/SignalP-6.0/>), demonstrating absence of canonical secretion signals.

**Supplementary Figure 3 Expression levels of the autophagy-secreted protein ENO1 in GBM cell lines**. **A-F** ENO1 expression significantly correlates with patient age, IDH mutation status, 1p19q co-deletion status, MGMT promoter status, and GBM subtype. **G** Analysis of ENO1 expression across all glioma cell lines in the Cancer Cell Line Encyclopedia (CCLE, <https://sites.broadinstitute.org/ccle>) database. **H** Western blot analysis of ENO1 expression in six GBM cell lines. **I** Western blot analysis of ENO1 knockdown efficiency in U87MG cells. **J** Western blot analysis of ENO1 overexpression in LN229 cells.

**Supplementary Figure 4 TLR4 knockdown attenuates ENO1-mediated oncogenic effects in GBM cells**. **A, B** Western blot validation of stable TLR4 knockdown using lentivirus-delivered shRNA. **C-F** Representative images and quantitative analysis of (C) colony formation, **(D)** proliferation, and migration/invasion **(E, F)** in ENO1 cells treated with or without TLR4 inhibition and cultured in ENO1-overexpressing conditioned medium (OE-ENO1 CM). Scale bars = 5 mm **(C)**, 100 μm **(D, E)**, and 500 μm **(F)**. Data represent mean ± SEM; ns: not significant, *P < 0.05, **P < 0.01, ***P < 0.001.

**Supplementary Figure 5** **Blockage of PI3K/AKT signaling pathway suppressed tumor growth in TMZ-treated xenograft mouse model**. **A** Diagram showing the process of establishing the orthotopic mouse model of GBM administration (n=6). **B** Representative bioluminescence images (BLI) from IVIS imaging showed the tumor luciferase fluorescence in the mouse xenografts of different treatment groups at days 7, 14, and 21. Saline control groups, *n* = 6; TMZ monotherapy groups, *n* = 6; TMZ + LY294002 (PI3K inhibitor) groups, *n* = 6; TMZ + LY294002 + TAK242 (TLR4 inhibitor) groups, *n* = 6. **C, D** Quantitative analysis of tumor growth using relative flux values (versus day 7) across treatment groups. **E** Kaplan–Meier curves showing the survival of GL261 xenograft-bearing mice in the different groups; *n* = 6 mice. **F** Representative IHC images showing the expression levels of ENO1, p-PI3K, and p-AKT in brain sections from each group. Scale bar, 100 μm. Data represent mean ± SEM; ns: not significant, *P < 0.05, **P < 0.01, ***P < 0.001.

**Supplementary Figure 6 Proteogenomic analysis of ENO1-associated signaling pathways in clinical GBM samples**. **A, B** Gene Set Variation Analysis (GSVA) and Gene Set Enrichment Analysis (GSEA) of clinical proteomic data from the Clinical Proteomic Tumor Analysis Consortium (CPTAC) database (<https://proteomics.cancer.gov/programs/cptac>).

**Supplementary Figure 7** Representative images and quantitative analysis of **(A)** colony formation, **(B)** proliferation, and migration/invasion **(C, D)** in GBM cells treated with or without SPHK1 inhibition PF-543 and cultured in rhENO1. Scale bars = 5 mm **(A)**, 100 μm **(B, D)**, and 500 μm **(C)**. Data represent mean ± SEM; ns: not significant, *P < 0.05, **P < 0.01, ***P < 0.001.

**Supplementary Figure 8 Analysis of single-cell RNA sequencing**. **A** The sequencing depth from 6 GBM samples. **B** Scatter plots demonstrating: Left: Relationship between transcript counts per cell versus gene counts per cell. Right: Relationship between transcript counts per cell versus mitochondrial gene expression percentage. **C** The cell types are identified by marker genes. **D** Pie chart analysis of cell populations associated with secretory signaling and cell-cell communication.

**Supplementary Figure 9 A** Heatmap showing the correlations of ENO1 expression with the expression of M2-associated marker genes in TCGA samples. **B** The TCGA database reveals a positive correlation between ENO1 expression and M2-associated marker **genes** (CD163, ARG1, TGFB2, IL-10, IL1R2 and MSR1) in gliomas.

**Supplementary Figure 10 Extracellular S1P induces M2 polarization of TAMs**. **A** qRT-PCR analysis of mRNA expression levels of M2-TAM markers (CD163, CD206, ARG1, and IL-10) following stimulation with 5 μM sphingosine-1-phosphate (S1P). **B** Immunofluorescence (IF) staining demonstrating protein expression of M2-TAM markers (CD68, CD163, and CD206) after treatment with 5 μM S1P. Scale bar = 10 μm. **C** IF staining showing protein expression of M2-TAM markers (CD163 and CD206) under treatment with varying concentrations of the SPHK1 inhibitor PF-543. Scale bar = 10 μm. **D** ELISA quantification of S1P levels in conditioned media from U87MG cells treated with different concentrations of the SPHK1 inhibitor PF-543. **E** qRT-PCR analysis of mRNA expression levels of M2-TAM markers (CD163, CD206, and ARG1) in M0 macrophages co-cultured with U87MG tumor cells and treated with 5 μM or 10 μM PF-543 (or vehicle control) for 24 h. **F** qRT-PCR quantification of M2-TAM marker (CD163, CD206, and ARG1) mRNA levels in M0 macrophages cultured for 24 h in conditioned media from U87MG cells treated with 5 μM or 10 μM PF-543 (or vehicle control). Data represent mean ± SEM; ns: not significant, *P < 0.05, **P < 0.01, ***P < 0.001.

**Supplementary Figure 11 A** Schematic representation of macrophage generation and recruitment. **B** ENO1 overexpression significantly enhances the recruitment of M0 macrophages. Scale bar = 50 μm. **C** ENO1 knockdown markedly reduces the recruitment of M0 macrophages. Scale bar = 50 μm. **D, E** Effect of conditioned medium from ENO1-overexpressing or ENO1-knockdown GBM cells on the secretion of IL-10, CCL18, and TGF-β by macrophages co-cultured with THP-1 cells. Data represent mean ± SEM; ns: not significant, *P < 0.05, **P < 0.01, ***P < 0.001.

**Supplementary Table Legends**

**Supplementary Table 1** Primers used for qRT-PCR.

**Supplementary Table 2** The sequences of shRNA.
