# Supplementary figures and images for: Autophagy-dependent secretion of ENO1 mediates chemoresistance of glioblastoma and tumor microenvironment remodeling
Source: Cell Death Dis. 2025 Dec 6;17(1):79. doi: 10.1038/s41419-025-08313-5 (PMC12827997; doi:10.1038/s41419-025-08313-5)

**A**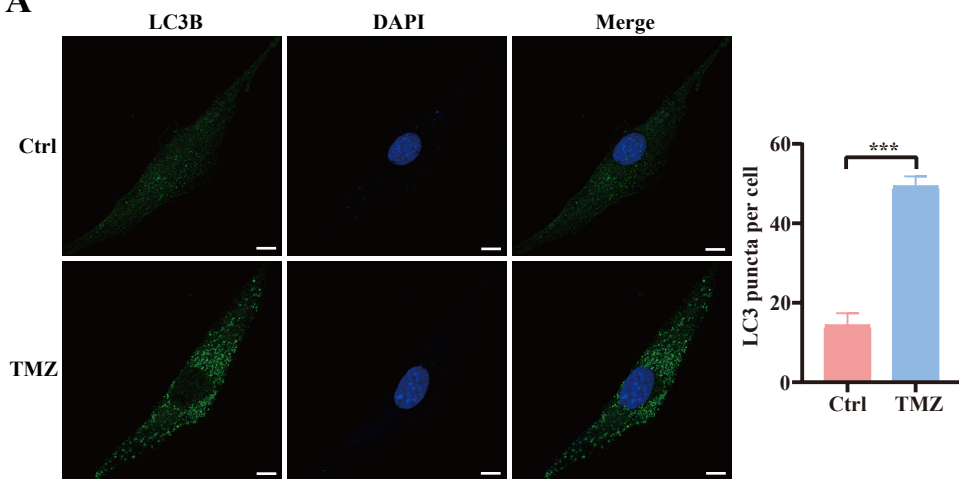**B**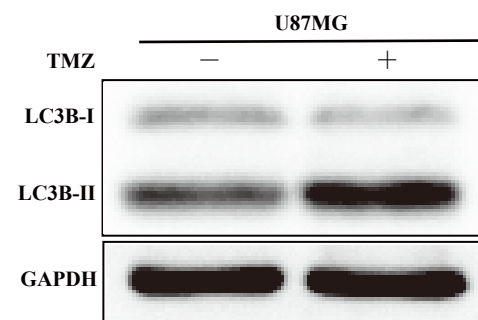**C**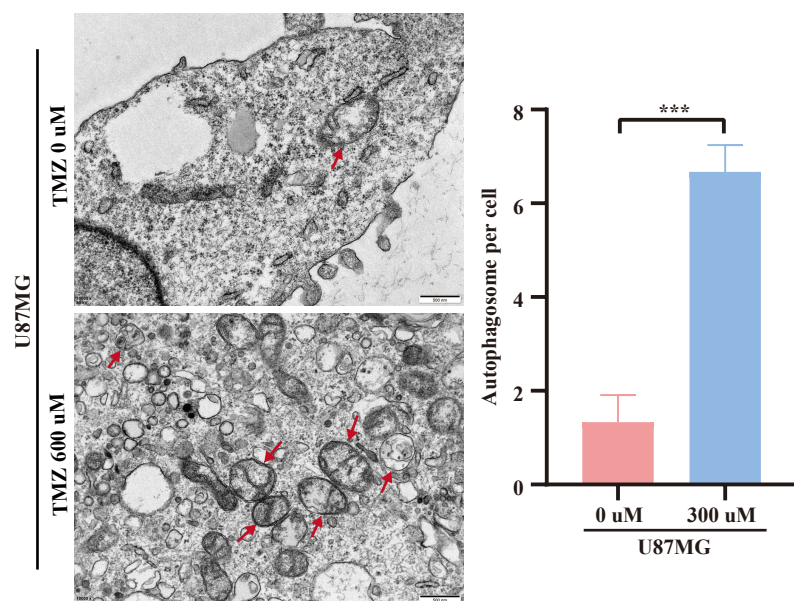**D**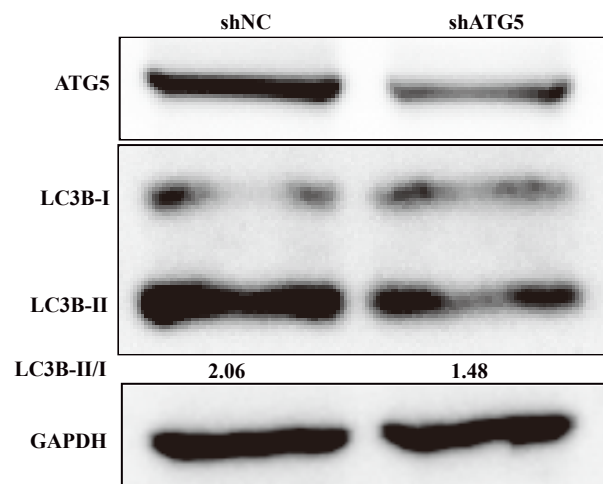**E**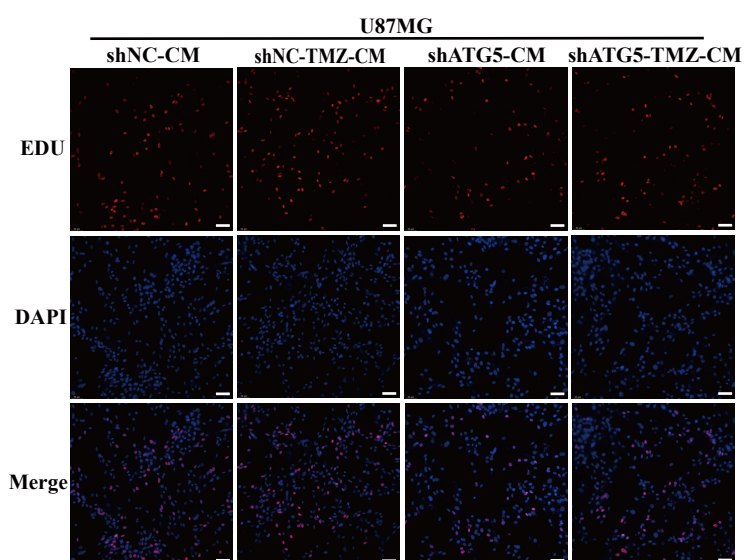**F**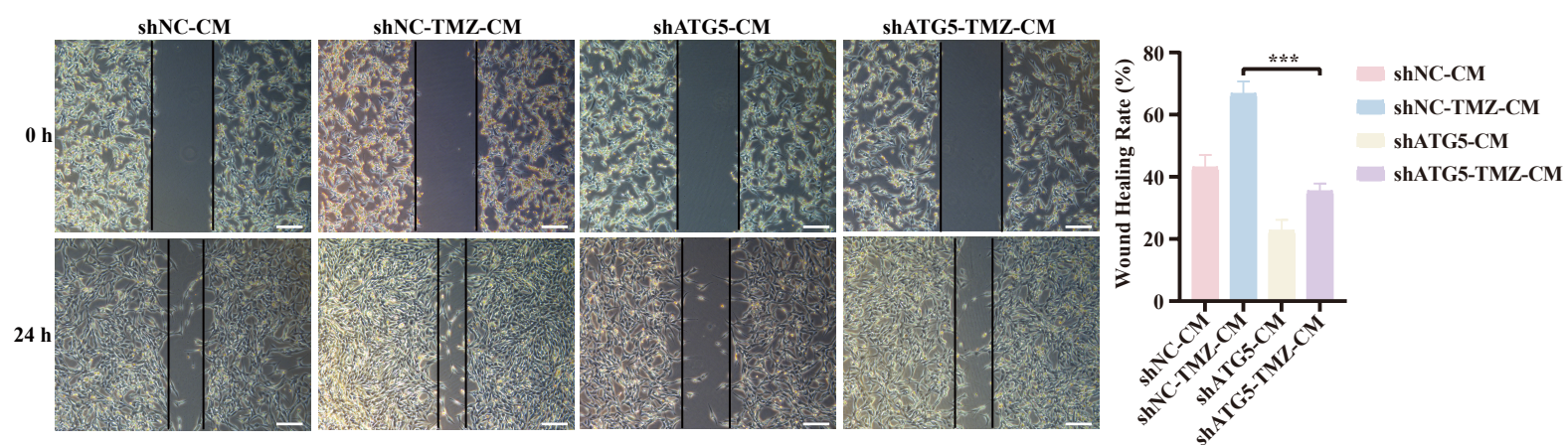

Supplement: Supplementary file 3 — Supplementary Figure 1 [file 41419_2025_8313_MOESM3_ESM.pdf]

A

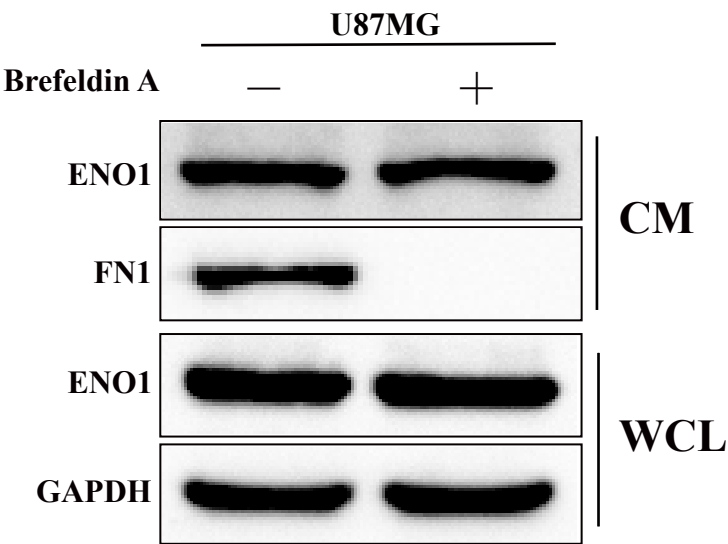

B

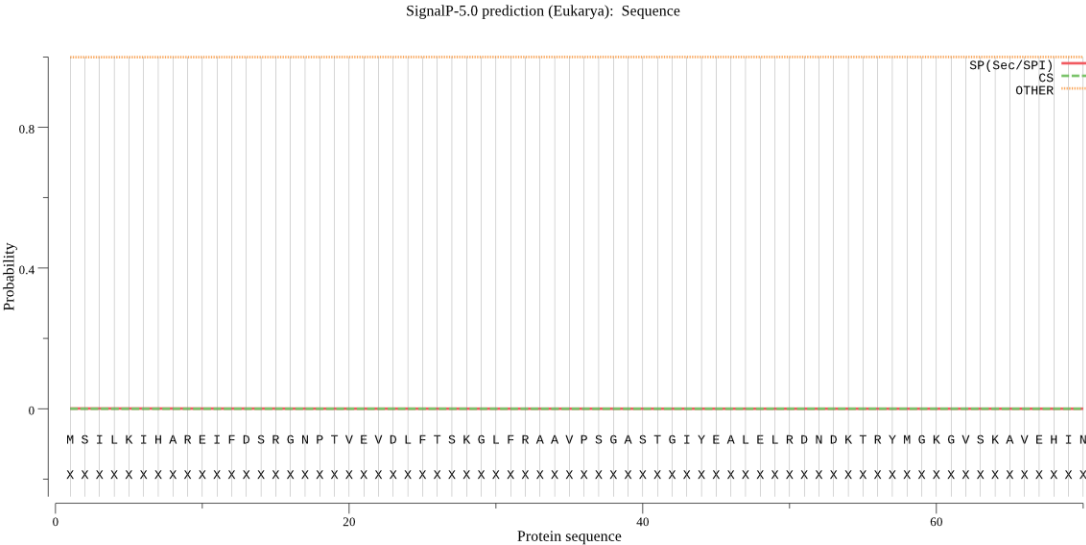

| Protein type | Signal Peptide (Sec/SPI) | Other  |
|--------------|--------------------------|--------|
| Likelihood   | 0.0006                   | 0.9994 |

Supplement: Supplementary file 4 — Supplementary Figure 2 [file 41419_2025_8313_MOESM4_ESM.pdf]

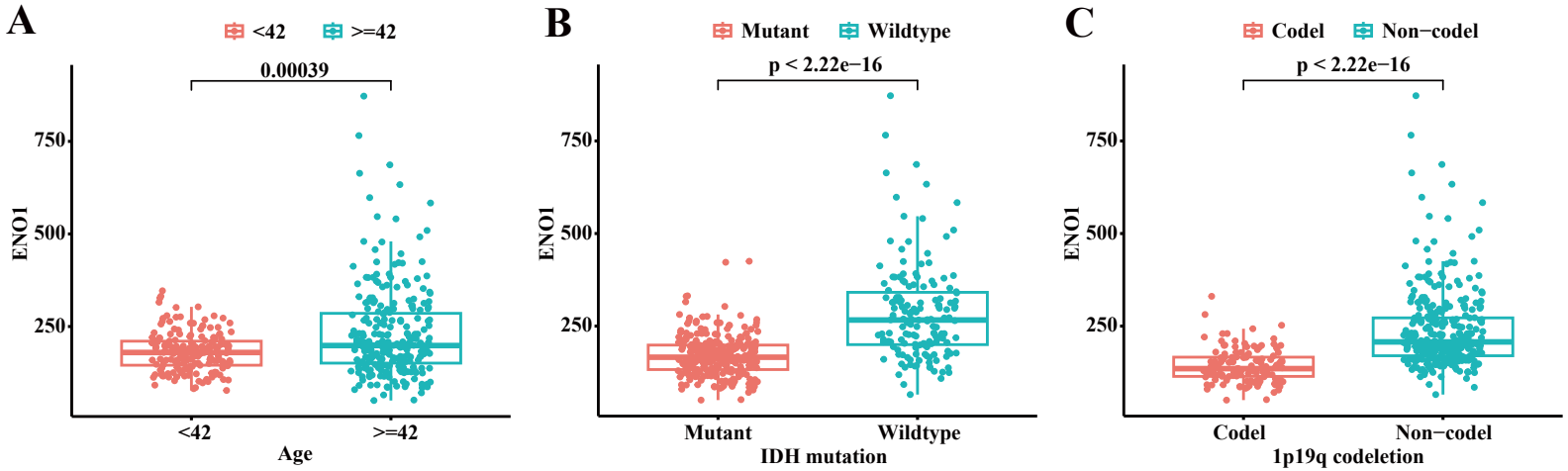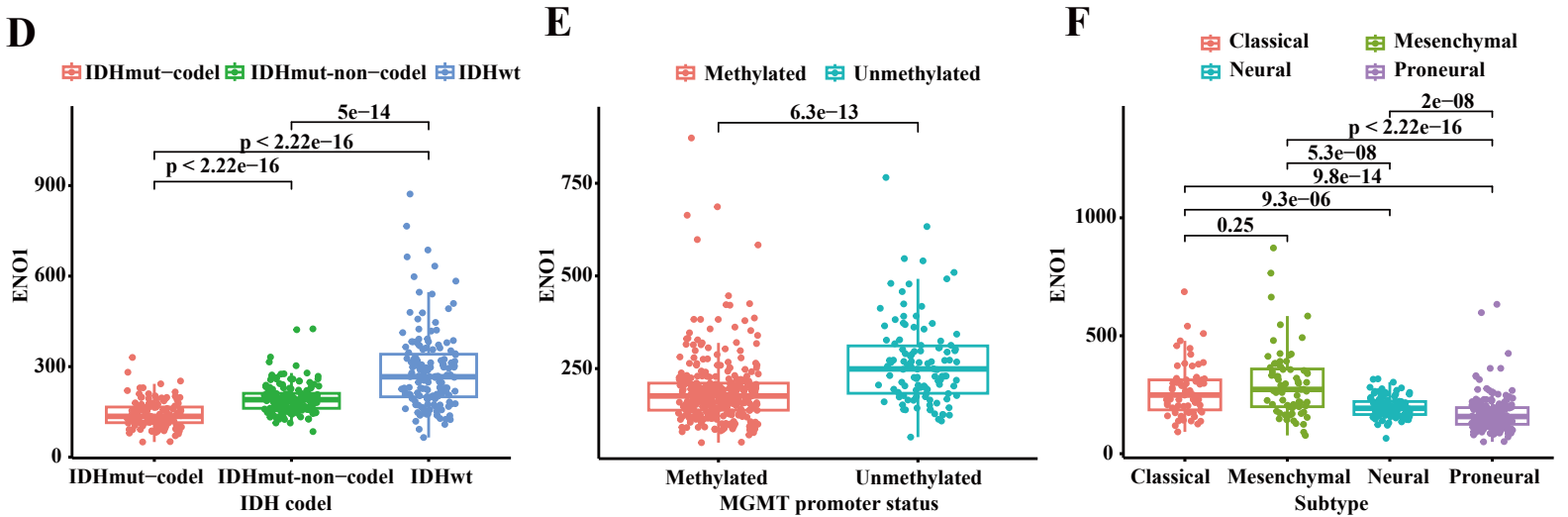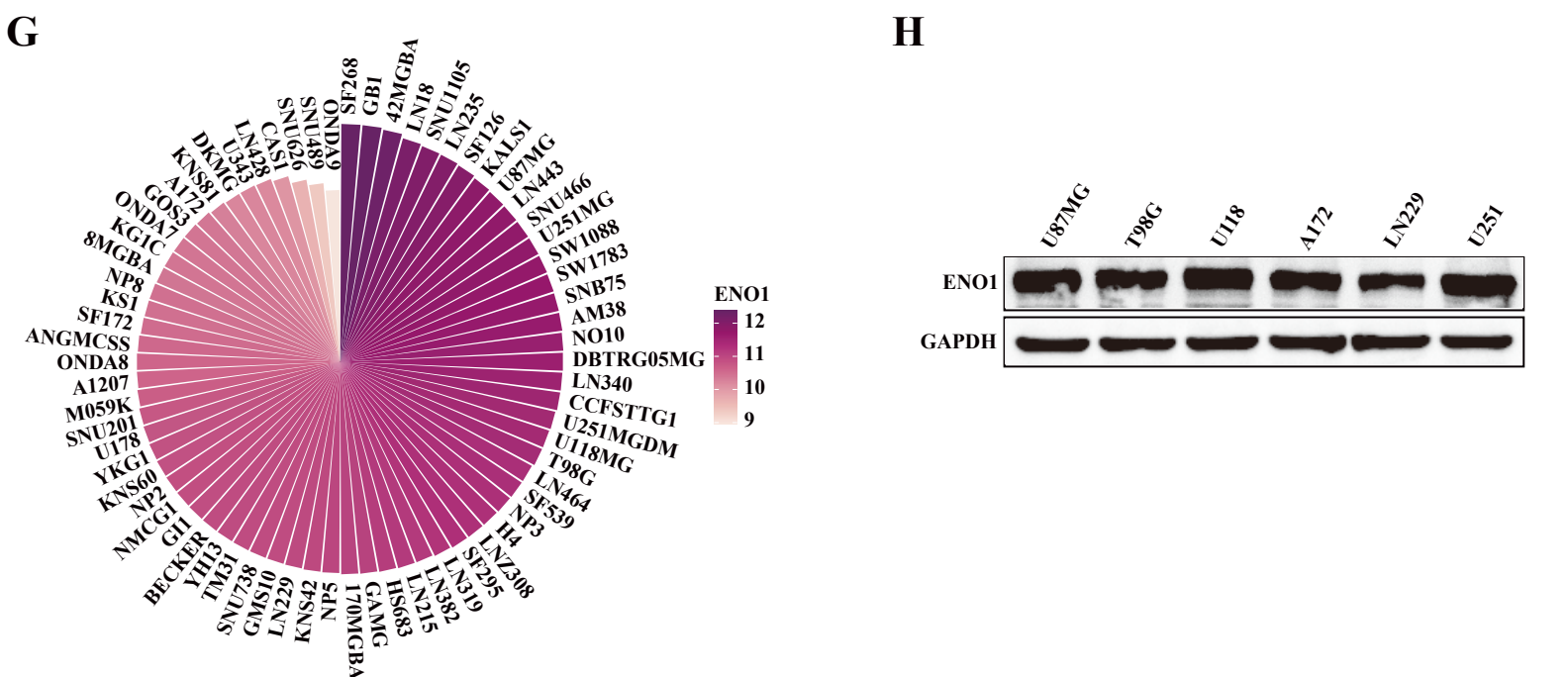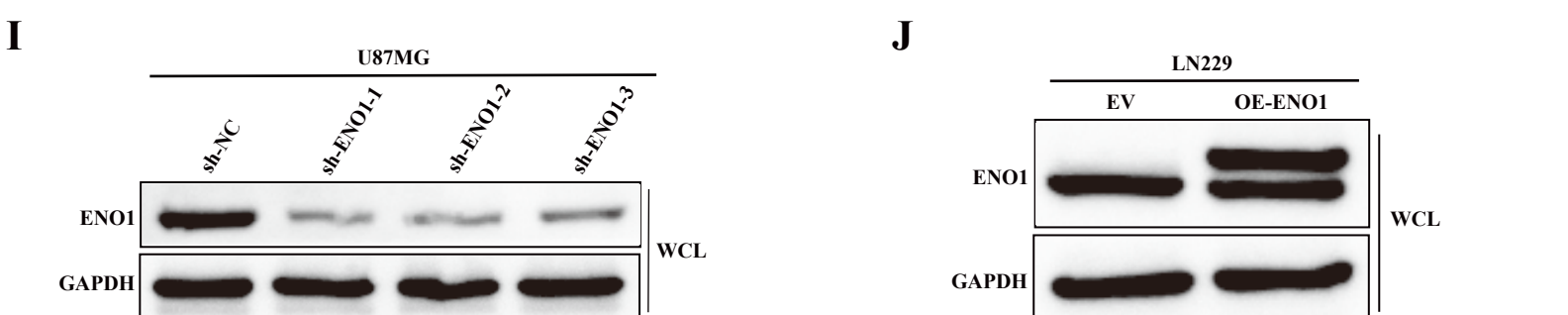

Supplement: Supplementary file 5 — Supplementary Figure 3 [file 41419_2025_8313_MOESM5_ESM.pdf]

**A**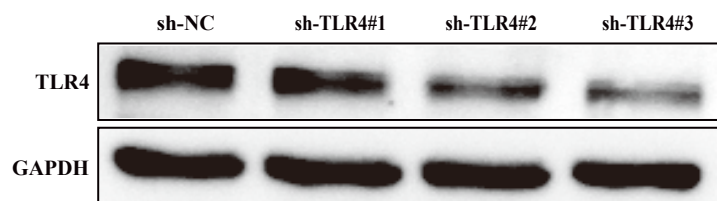**B**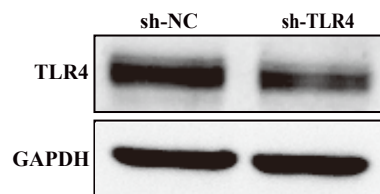**C**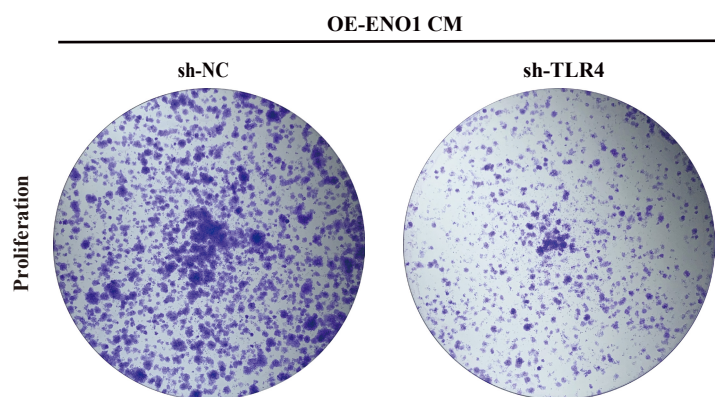**D**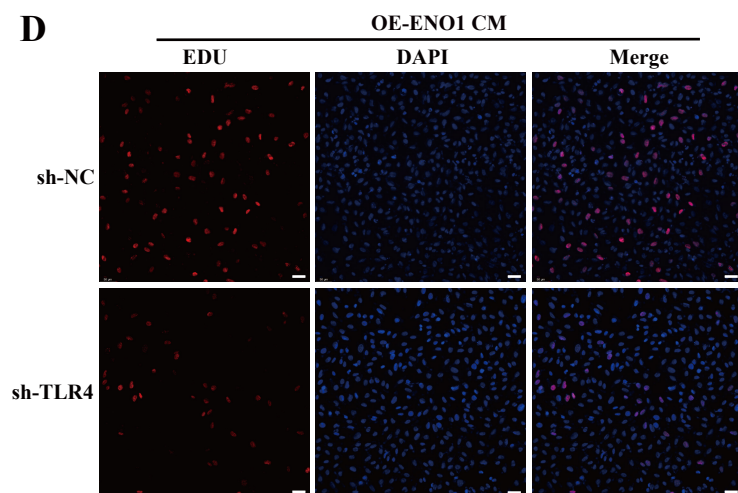**E**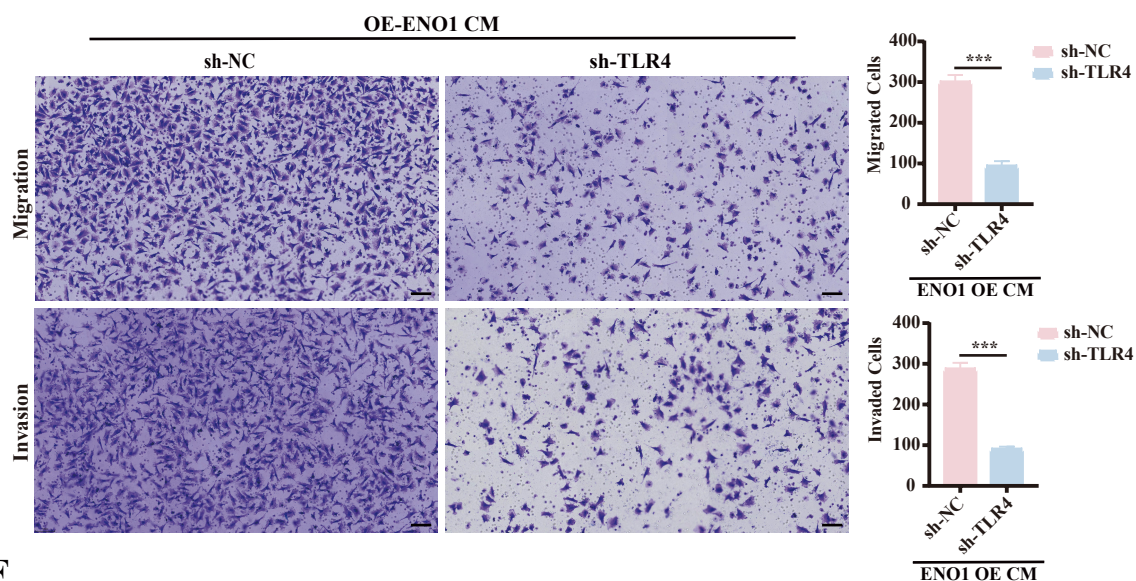**F**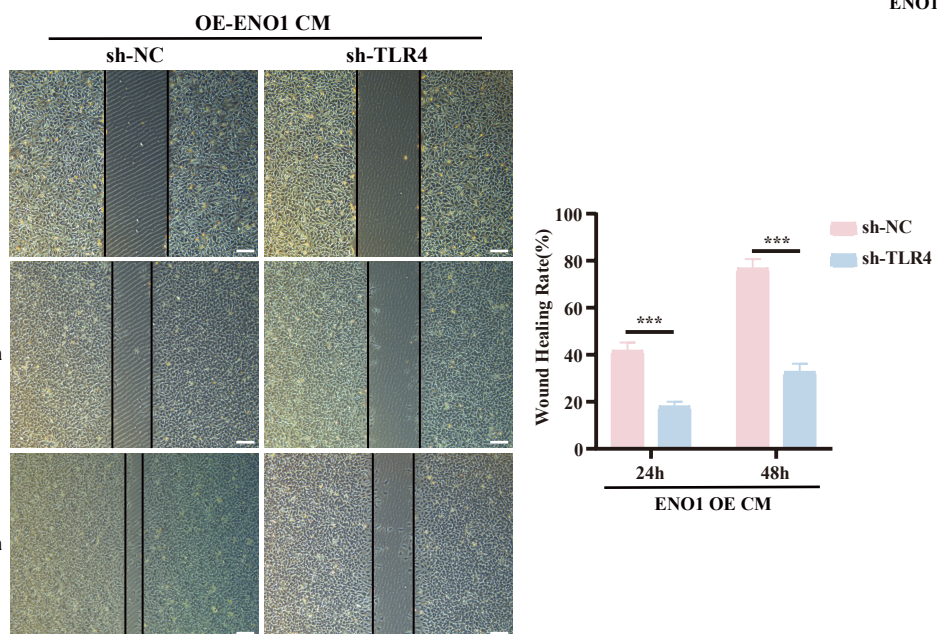

Supplement: Supplementary file 6 — Supplementary Figure 4 [file 41419_2025_8313_MOESM6_ESM.pdf]

**A**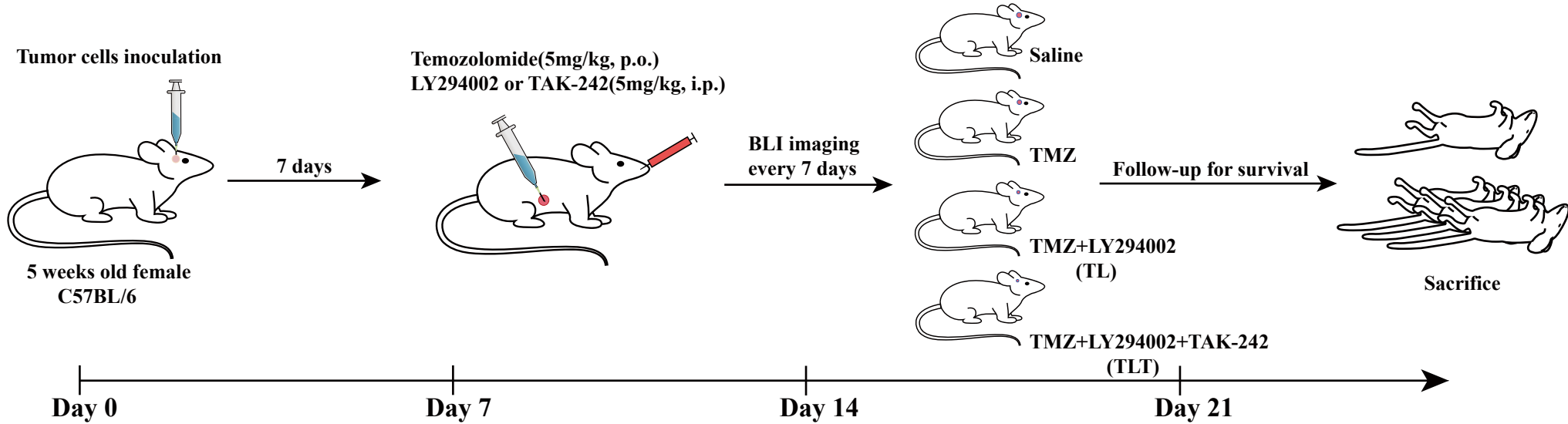**B**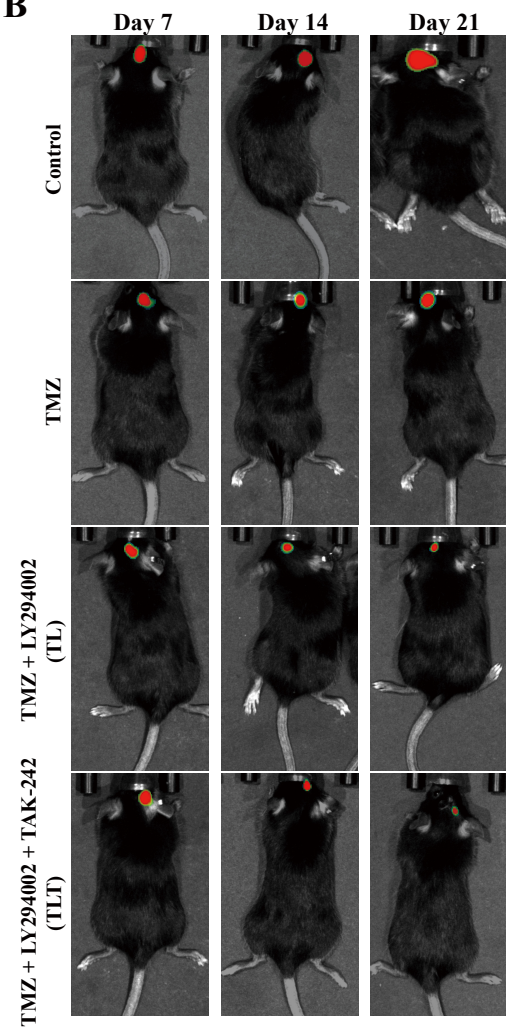**C**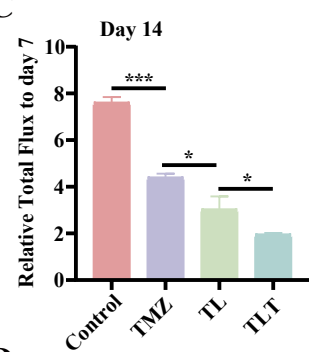**D**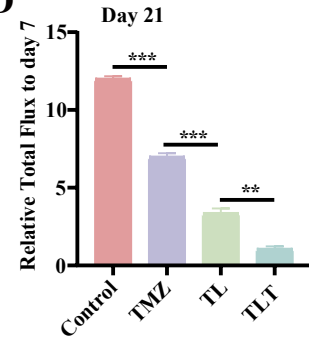**E**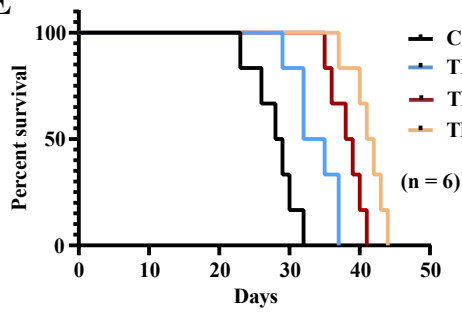**F**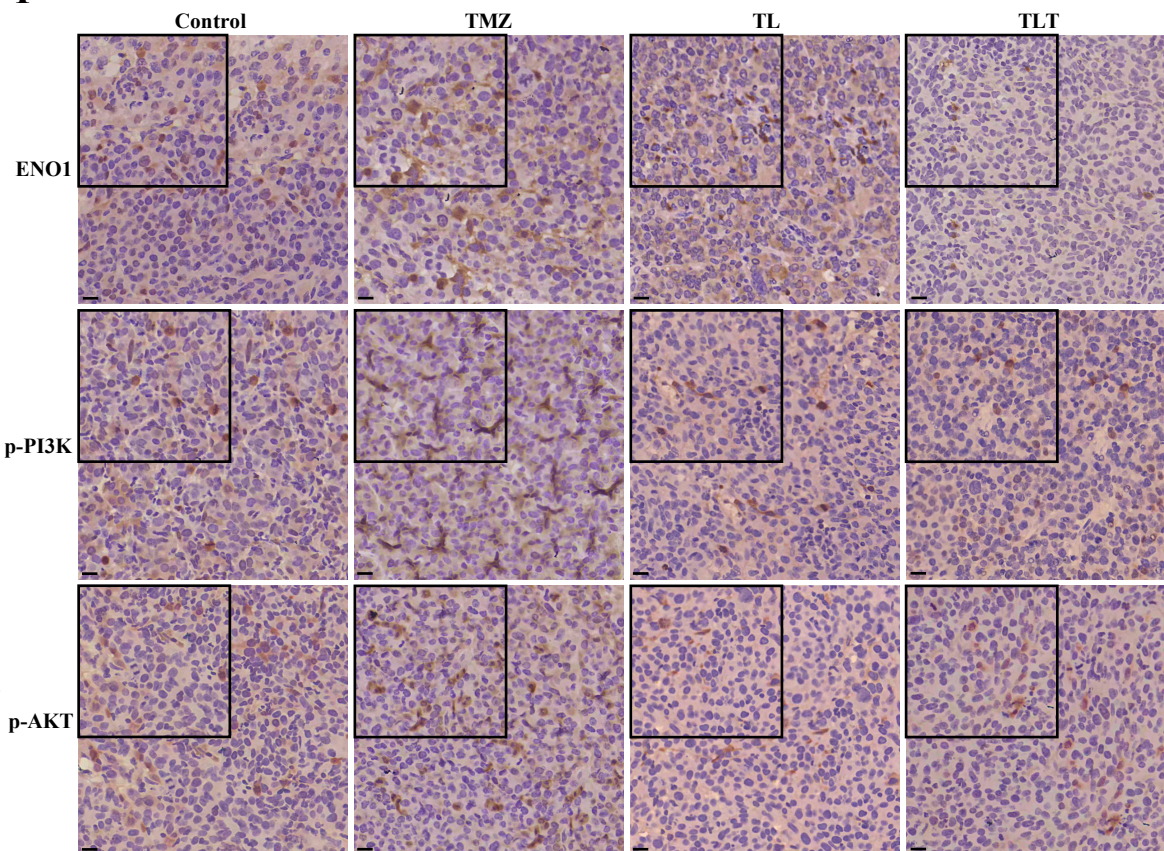

Supplement: Supplementary file 7 — Supplementary Figure 5 [file 41419_2025_8313_MOESM7_ESM.pdf]

A

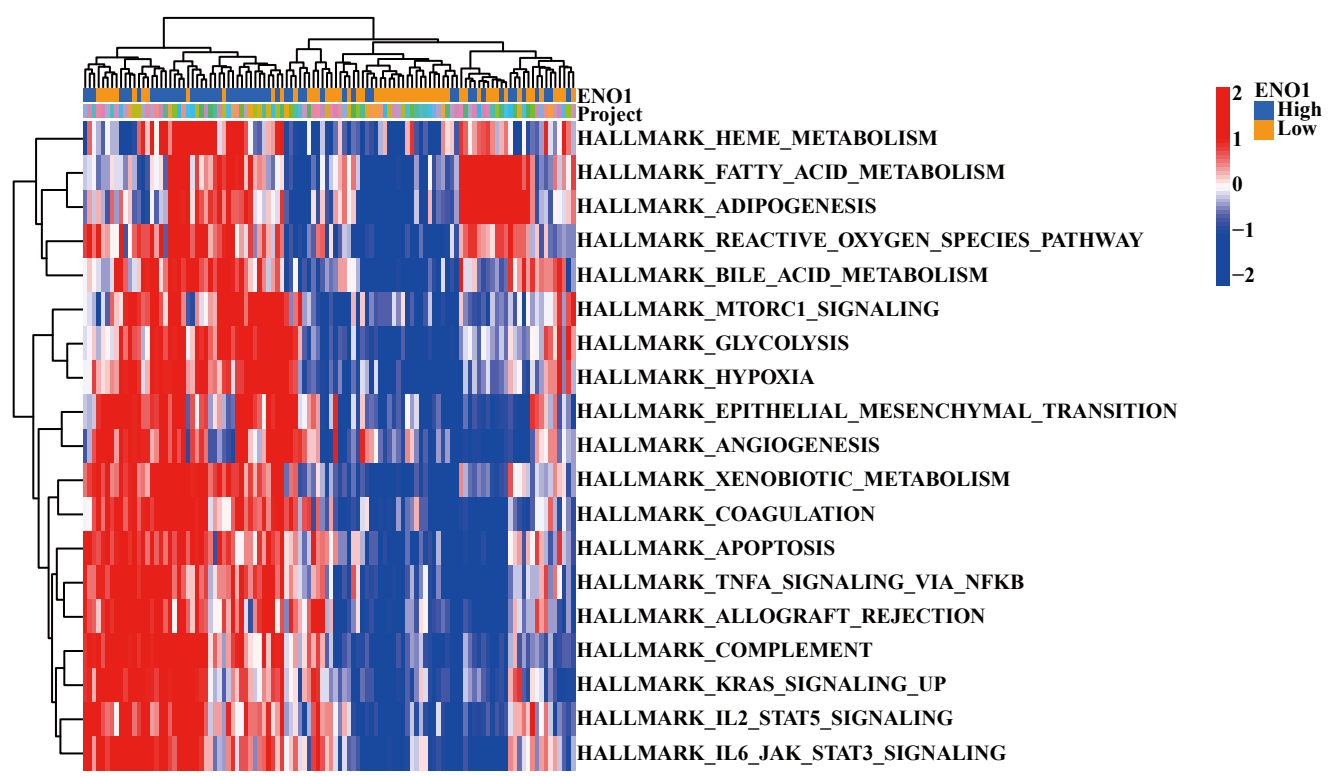

B

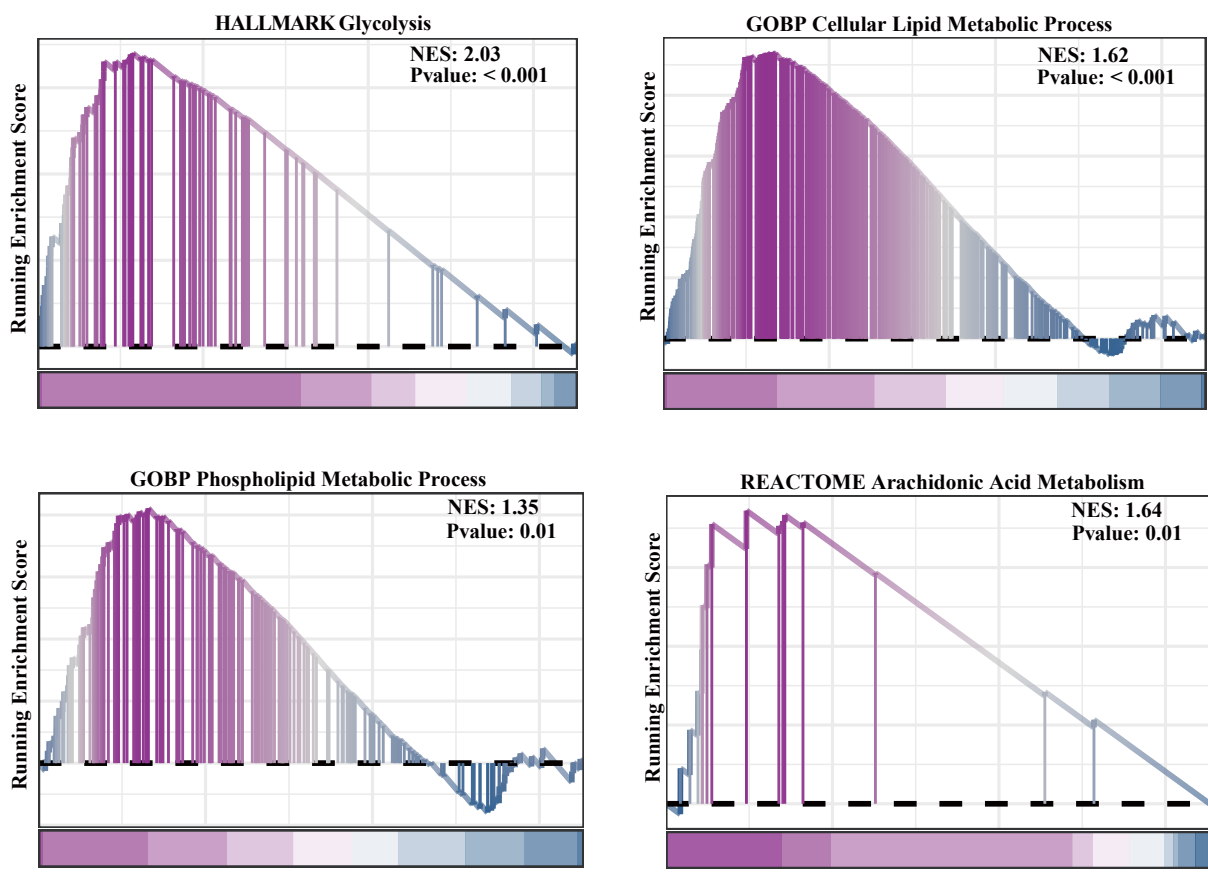

Supplement: Supplementary file 8 — Supplementary Figure 6 [file 41419_2025_8313_MOESM8_ESM.pdf]

**A**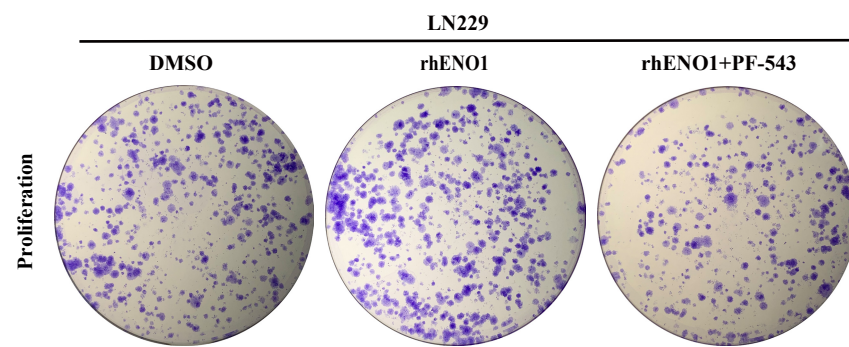**B**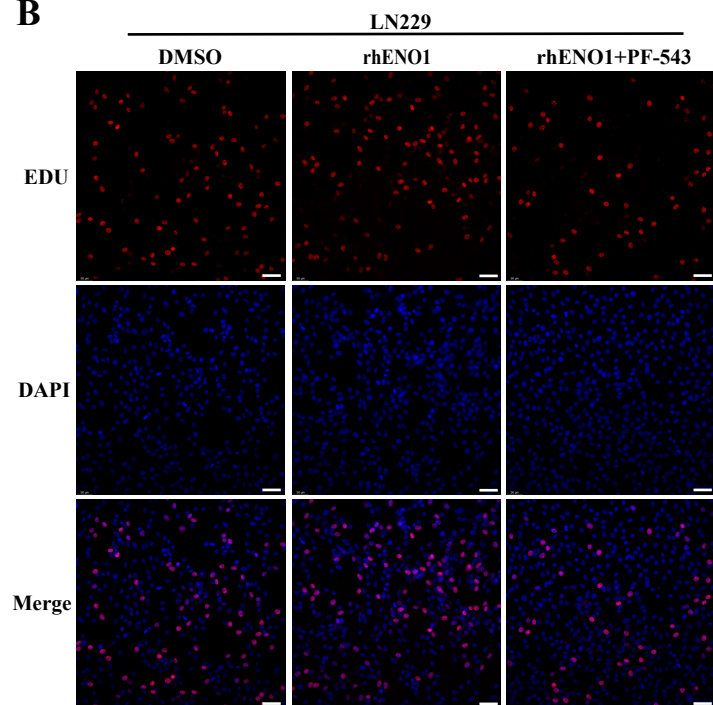**C**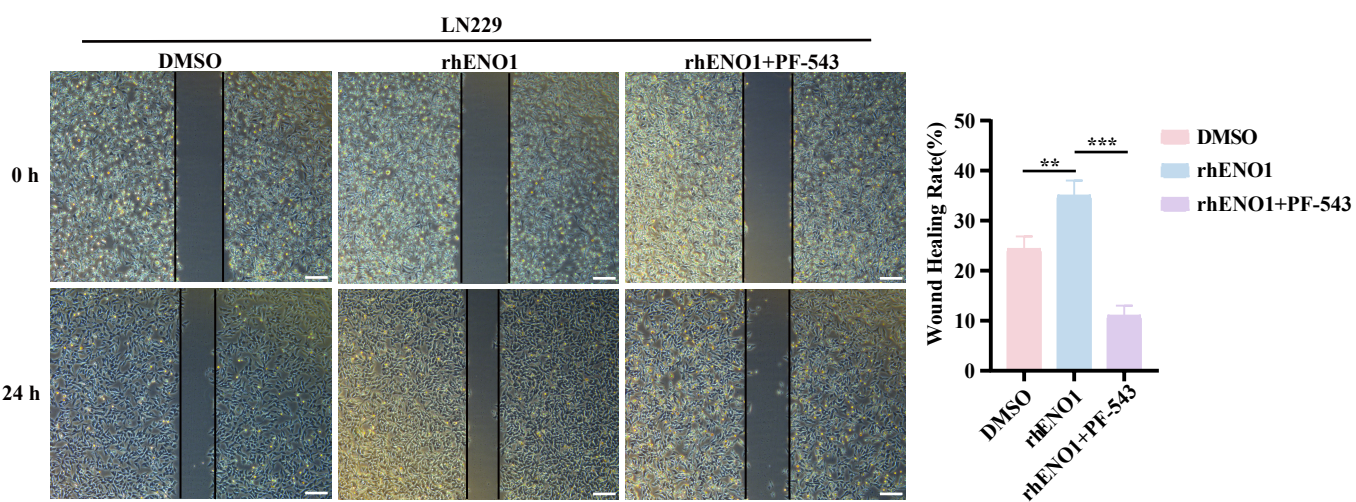**D**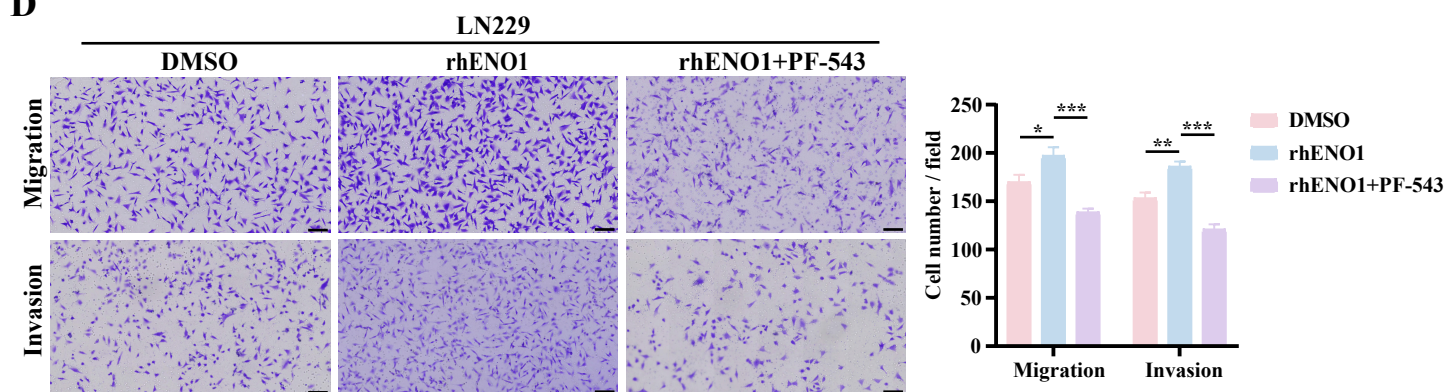

Supplement: Supplementary file 9 — Supplementary Figure 7 [file 41419_2025_8313_MOESM9_ESM.pdf]

A

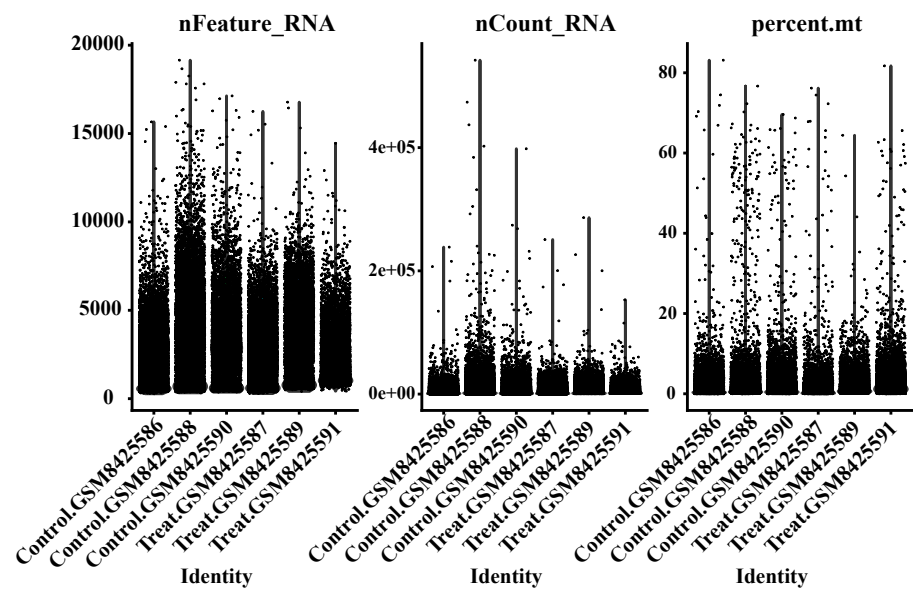

B

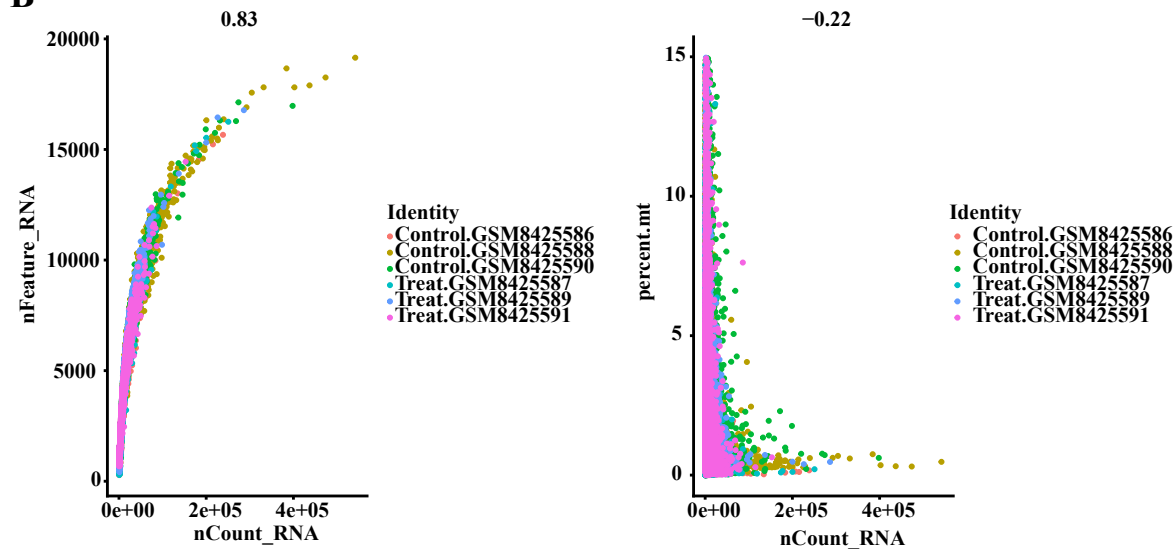

C

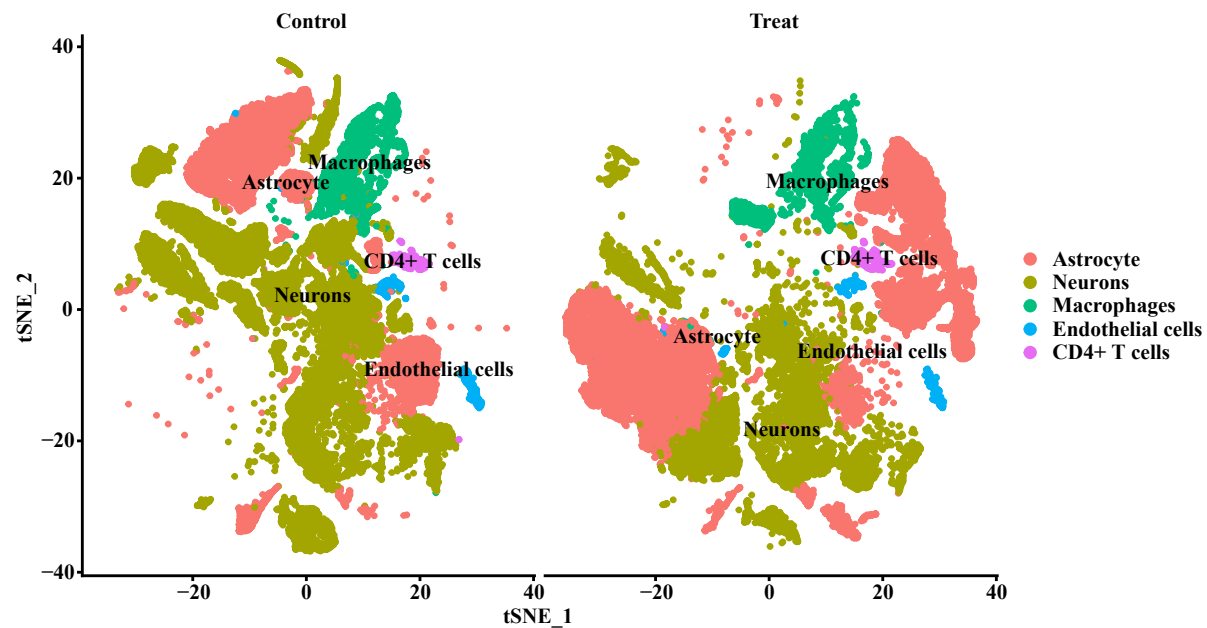

D

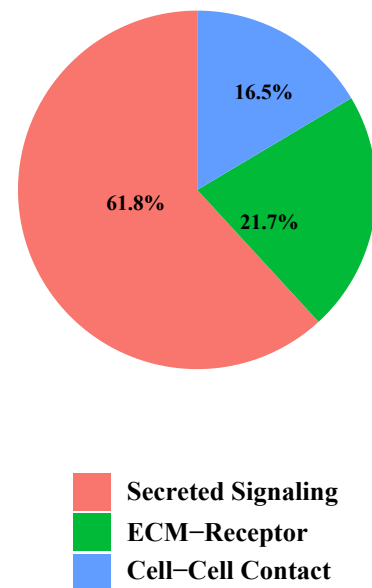

Supplement: Supplementary file 10 — Supplementary Figure 8 [file 41419_2025_8313_MOESM10_ESM.pdf]

A

Correlation Heatmap between ENO1 expression and M2 markers genes

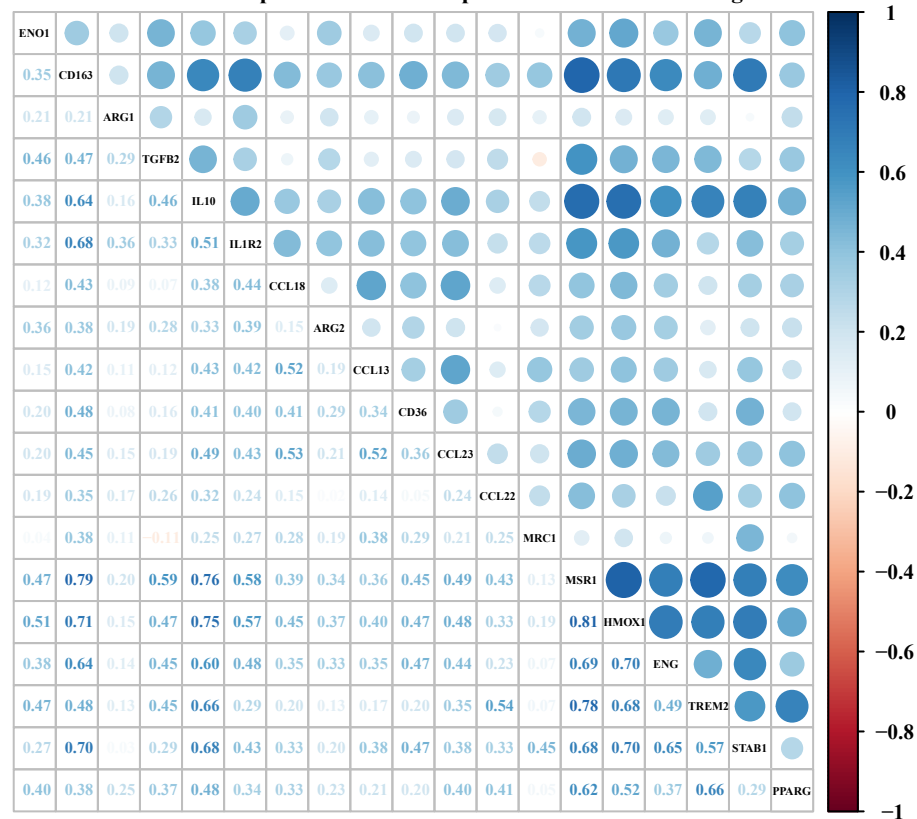

B

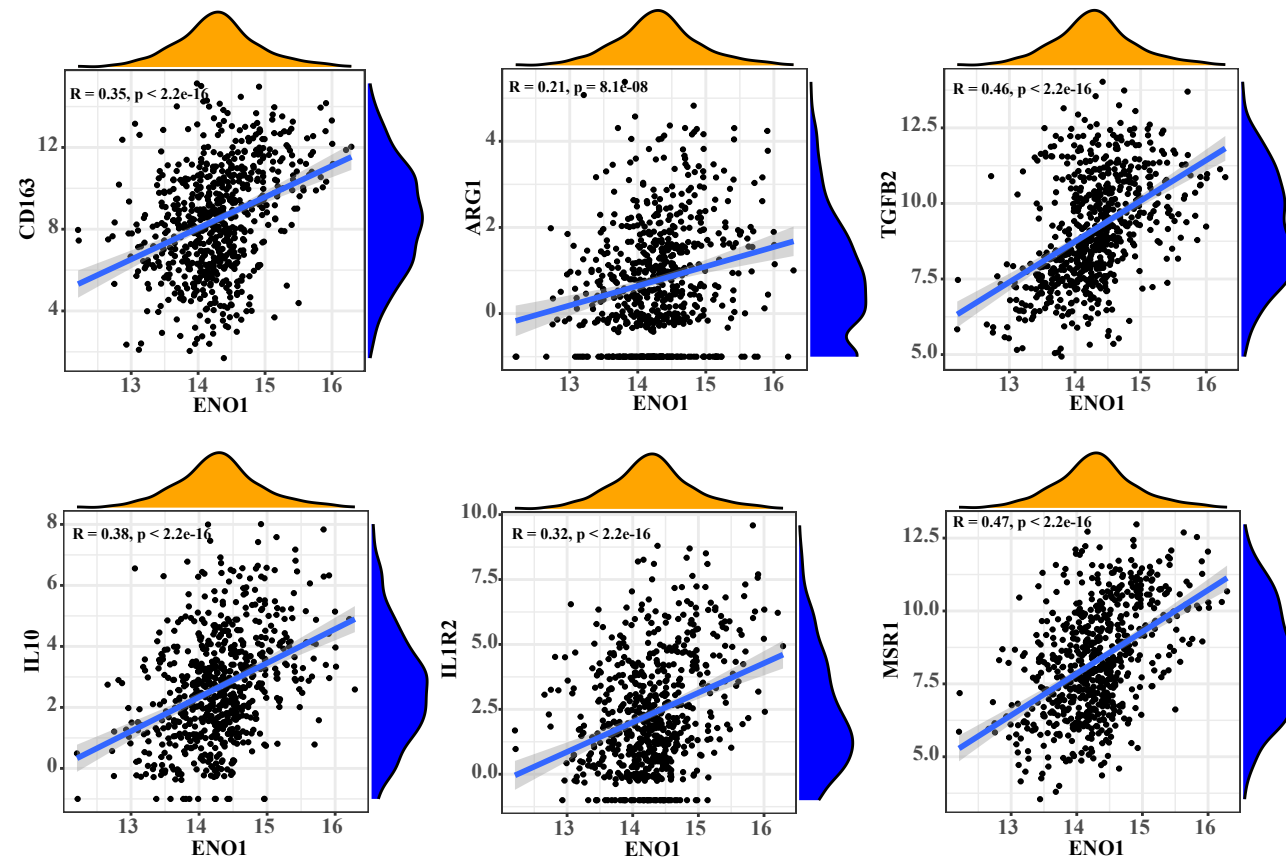

Supplement: Supplementary file 11 — Supplementary Figure 9 [file 41419_2025_8313_MOESM11_ESM.pdf]

**A**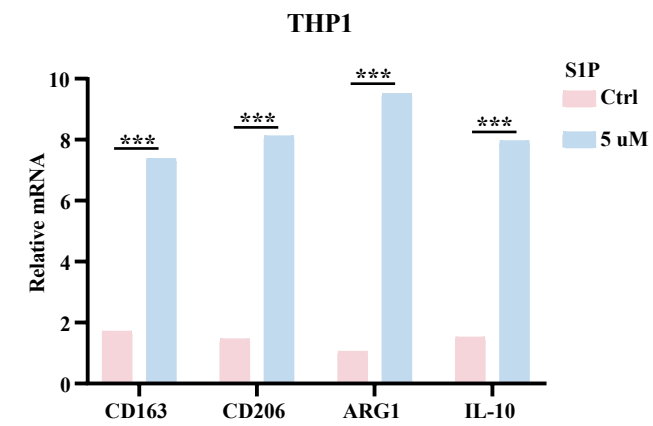**B**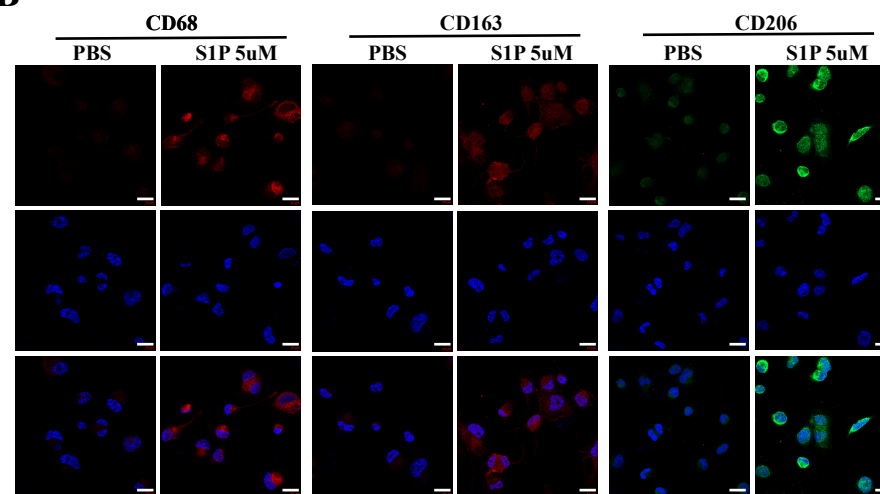**C**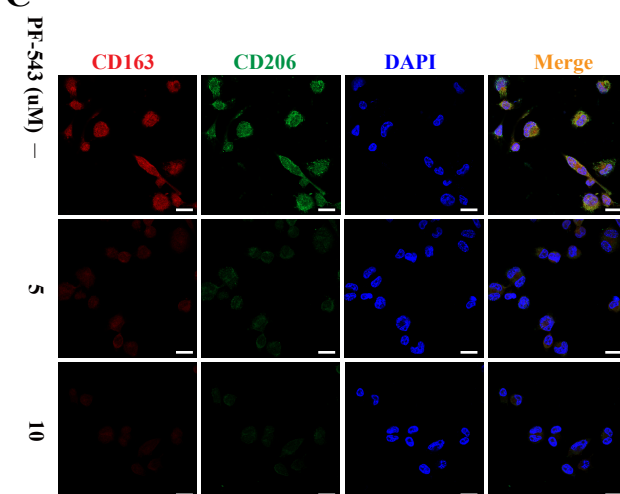**D**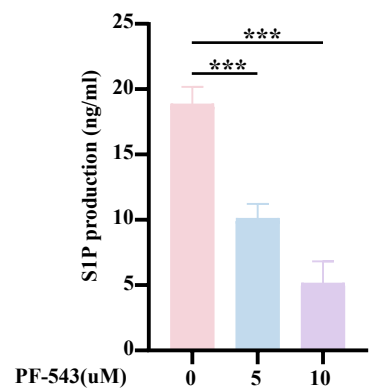**E**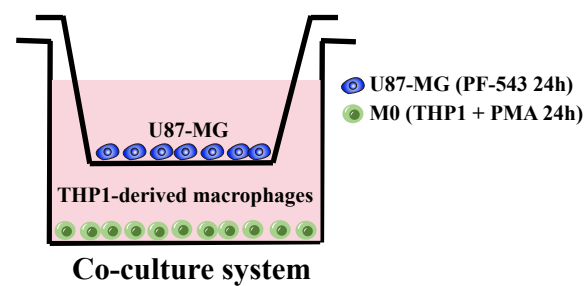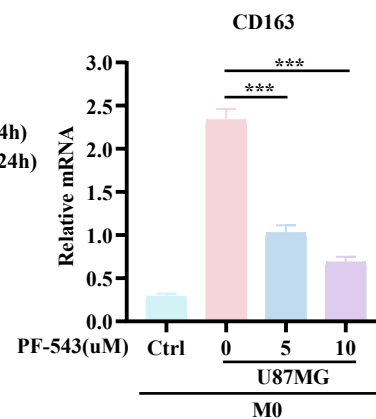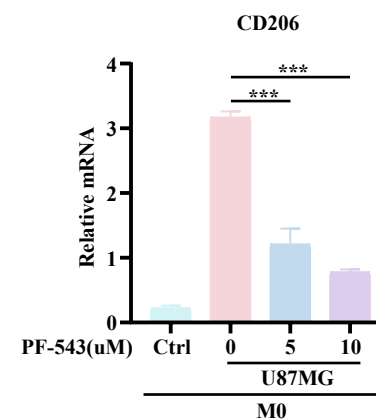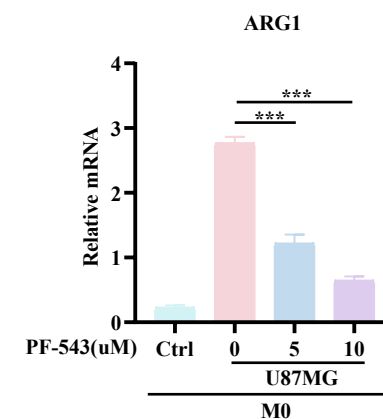**F**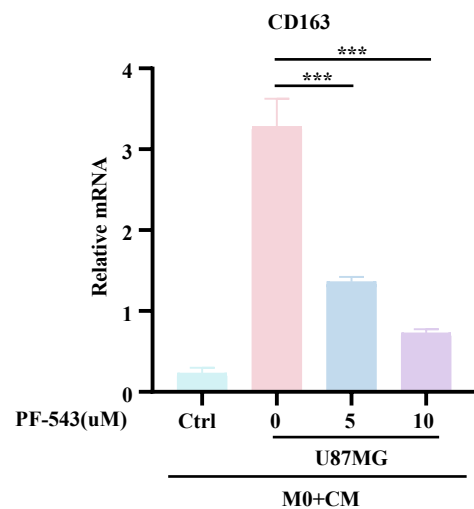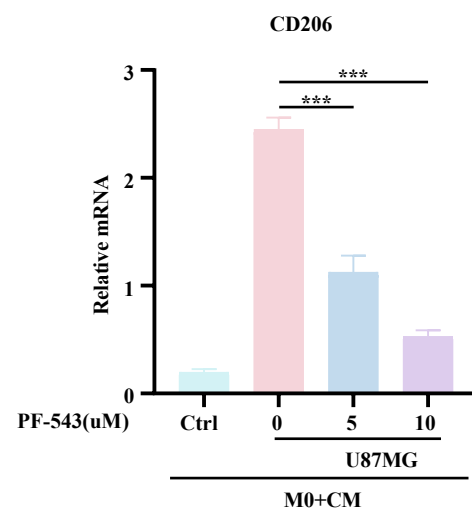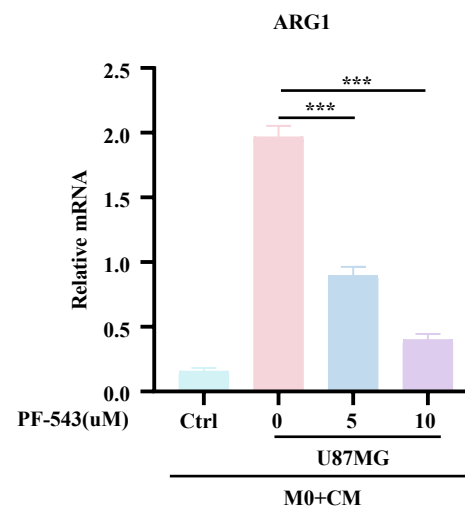

Supplement: Supplementary file 12 — Supplementary Figure 10 [file 41419_2025_8313_MOESM12_ESM.pdf]

**A**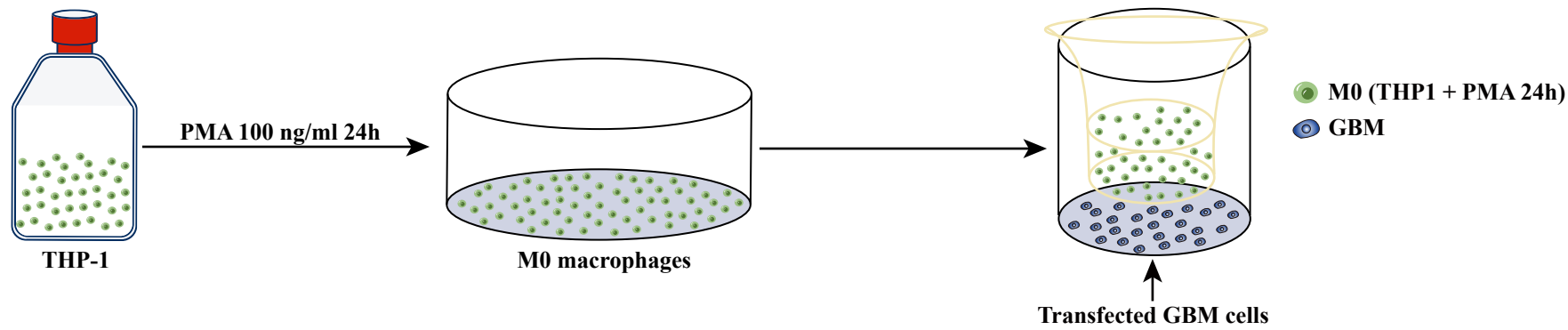**B**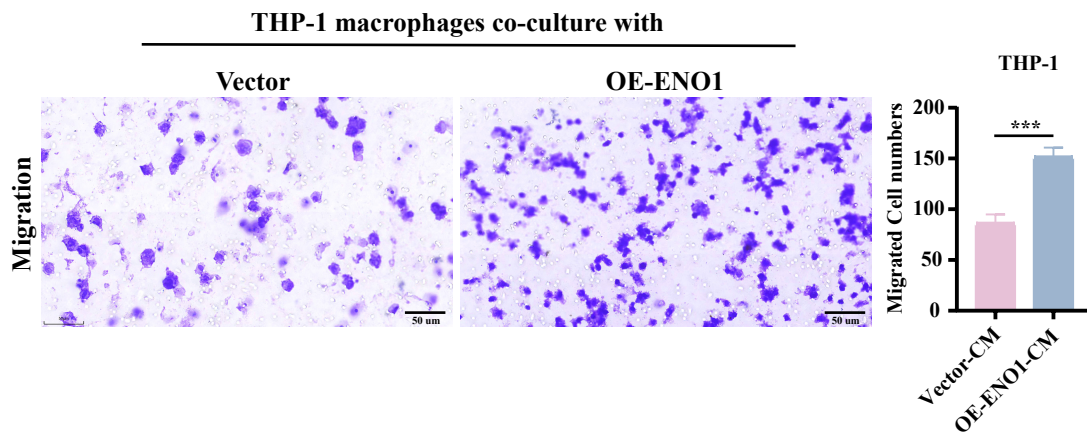**C**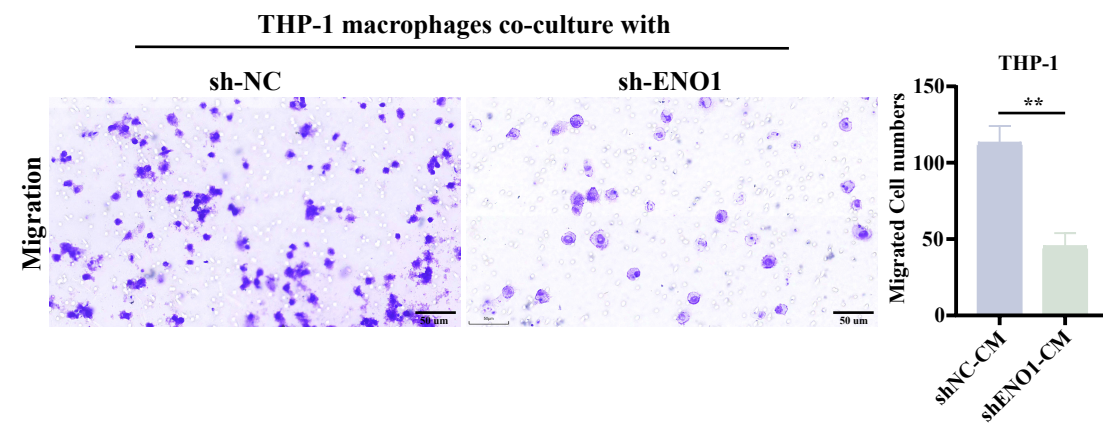**D**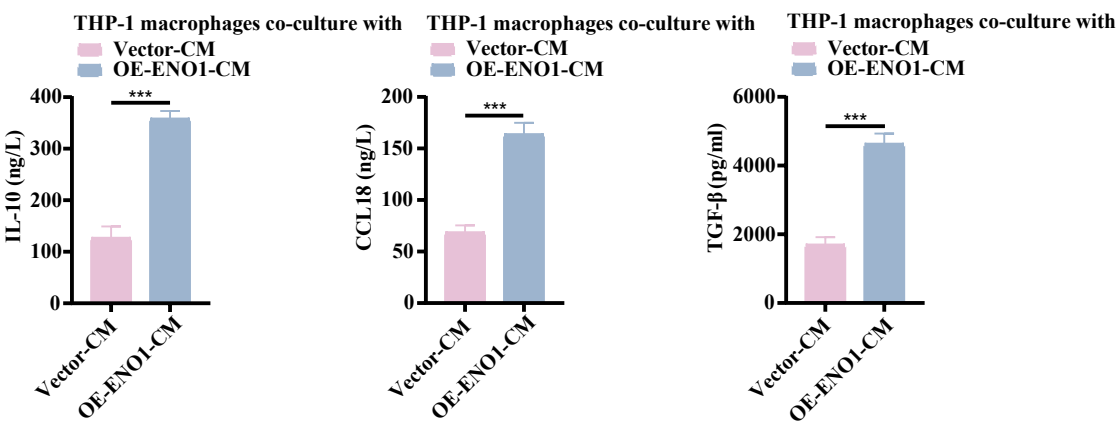**E**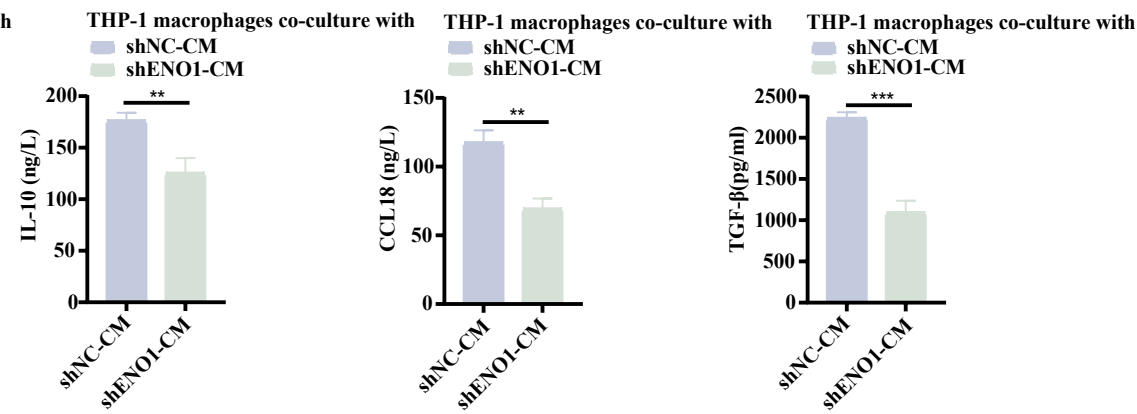

Supplement: Supplementary file 13 — Supplementary Figure 11 [file 41419_2025_8313_MOESM13_ESM.pdf]
