# Supplementary material for: Autophagy-dependent secretion of ENO1 mediates chemoresistance of glioblastoma and tumor microenvironment remodeling
Source: Cell Death Dis. 2025 Dec 6;17(1):79. doi: 10.1038/s41419-025-08313-5 (PMC12827997; doi:10.1038/s41419-025-08313-5)
Supplement: Supplementary file 14 — Supplementary Table [file 41419_2025_8313_MOESM14_ESM.pdf]

Supplementary Table 1 Primers used for qRT-PCR.

Gene name	Forward Primer Sequence (5' - 3')	Reverse Primer Sequence (5' - 3')
CD163	TTTGTCAACTTGAGTCCCTTCAC	TCCCGCTACACTTGTTTTCAC
CD206	CGATCCGACCCTTCCTTGAC	TGTCTCCGCTTCATGCCATT
ARG1	TCATCTGGGTGGATGCTCACAC	GAGAATCCTGGCACATCGGGAA
IL-10	GGCGCTGTCATCGATTTCTTC	GCCACCCTGATGTCTCAGTT
GAPDH	GGCATGGACTGTGGTCATGAG	TGCACCACCAACTGCTTAGC

Supplementary Table 2 The sequences of shRNA.

Vector	Sequence (5' to 3')
sh*ATG5*-1	GCAACTCCTCCAAGATCTA
sh*ATG5*-2	GCTGAAGAAATGGTTCTAA
sh*ATG5*-3	TGCCTTGTTCTAACTCCAATT
Negative Control	TTCTCCGAACGTGTCACGT

Vector	Sequence (5' to 3')
sh*ENO1*-1	GAATGTCATCAAGGAGAAATA
sh*ENO1*-2	CGTGAACGAGAAGTCCTGCAA
sh*ENO1*-3	CGCATTGGAGCAGAGGTTTAC
Negative Control	CCTAAGGTTAAGTCGCCCTCG

Vector	Sequence (5' to 3')
sh*TLR4*-1	AGACTACTACCTCGATGATAT
sh*TLR4*-2	CCCTGCTGGATGGTAAATCAT
sh*TLR4*-3	GGACCUCUCUCAGUGUCAATT
Negative Control	UUCUCCGAACGUGUCACGUTT
